# Powerful testing via hierarchical linkage disequilibrium in haplotype association studies

**DOI:** 10.1002/bimj.201800053

**Published:** 2019-01-28

**Authors:** Brunilda Balliu, Jeanine J. Houwing‐Duistermaat, Stefan Böhringer

**Affiliations:** ^1^ Department of Biomathematics David Geffen School of Medicine UCLA Los Angeles CA USA; ^2^ School of Mathematics University of Leeds Leeds UK; ^3^ Department of Biomedical Data Sciences Section Medical Statistics and Bioinformatics Leiden University Medical Center Leiden The Netherlands

**Keywords:** cis interactions, genome‐wide association study, haplotype association study, linkage disequilibrium

## Abstract

Marginal tests based on individual SNPs are routinely used in genetic association studies. Studies have shown that haplotype‐based methods may provide more power in disease mapping than methods based on single markers when, for example, multiple disease‐susceptibility variants occur within the same gene. A limitation of haplotype‐based methods is that the number of parameters increases exponentially with the number of SNPs, inducing a commensurate increase in the degrees of freedom and weakening the power to detect associations. To address this limitation, we introduce a hierarchical linkage disequilibrium model for disease mapping, based on a reparametrization of the multinomial haplotype distribution, where every parameter corresponds to the cumulant of each possible subset of a set of loci. This hierarchy present in the parameters enables us to employ flexible testing strategies over a range of parameter sets: from standard single SNP analyses through the full haplotype distribution tests, reducing degrees of freedom and increasing the power to detect associations. We show via extensive simulations that our approach maintains the type I error at nominal level and has increased power under many realistic scenarios, as compared to single SNP and standard haplotype‐based studies. To evaluate the performance of our proposed methodology in real data, we analyze genome‐wide data from the Wellcome Trust Case‐Control Consortium.

## 1 INTRODUCTION

Marginal tests based on individual single nucleotide polymorphisms (SNPs) have dominated association analyses in the past decade. Although single SNP analyses have led to the identification of hundreds of genetic variants associated with many complex diseases (Hindorff et al., 2009), greater power might be achieved by using haplotype‐based approaches, analyzing multiple markers simultaneously. Haplotype‐based association methods incorporate linkage disequilibrium (LD) information from multiple markers and can be more powerful for gene mapping than methods based on single SNPs (Akey, Jin, & Xiong, 2001; Epstein & Satten, 2003; Zaykin et al., 2002). For example, haplotype‐based methods will be more powerful when multiple disease‐susceptibility variants, each with an independent effect, occur within the same gene (Morris & Kaplan, 2002). Moreover, haplotype‐based methods could be preferable to single SNP‐based association methods when diseases arise from the interaction of multiple cis‐acting susceptibility variants found within a gene, forming a “super‐allele” (Clark et al., 1998; Drysdale et al., 2000; Hollox et al., 2001; Joosten, Toepoel, Mariman, & Van Zoelen, 2001; Tavtigian et al., 2001), since haplotype‐based methods allow for super‐additivity of multiple genetic variants, whereas marginal tests do not (Epstein & Satten, 2003).

Standard haplotype association methods test for differences in haplotype distributions between cases and controls or perform regression analyses in which haplotypes are treated as categorical variables (Boehringer & Pfeiffer, 2009; Epstein & Satten, 2003; Lin & Zeng, 2006; Schaid, Rowland, Tines, Jacobson, & Poland, 2002; Spinka, Carroll, & Chatterjee, 2005; Zaykin et al., 2002). Two detailed reviews on existing methods for haplotype‐based association analysis are provided by Schaid (2004) and Liu, Zhang, and Zhao (2008). Moving from single‐SNP to haplotype‐based analyses results in a considerable increase in polymorphism and in a commensurate increase in the number of association parameters and therefore the degrees of freedom (df) of the association tests. As a result, the global score or likelihood ratio test statistics will be weakly powered. Moreover, when the haplotype data is sparse, the χ^2^ approximation of the distribution of the test statistics might be invalid. An additional difficulty is the ambiguity in haplotype phase when only genotype data are observed. Ambiguity can be handled using an expectation‐maximization (EM) algorithm (Dempster, Laird, & Rubin, 1977; Excoffier & Slatkin, 1995), however, the additional assumption of Hardy–Weinberg equilibrium (HWE) is needed. The df problem and the problem due to many rare haplotypes remain a limitation and force to employ heuristic methods, such as grouping of rare haplotypes (Schaid, 2004). Due to these limitations of the haplotype‐based methods and the myriad possible genetic architectures of complex human diseases, the relative efficiency of using haplotypes versus single markers remains largely unexplored and is often decided by practical considerations.

In this work, we introduce a hierarchical LD model for trait mapping that enables us to employ flexible testing strategies over a range of parameter sets: from standard single SNP analyses through the comparison of full haplotype distributions, thereby allowing to reduce df and increase the power to detect associations. Hierarchical LD has been previously defined (Geiringer, 1944; Gorelick & Laubichler, 2004; Lewontin & others, 1974; Lou et al., 2003; Weir, 1990). Geiringer (1944); Gorelick and Laubichler (2004) give the same parametrization that we use but it is derived differently. Lewontin and others (1974); Lou et al. (2003); Weir (1990) consider special cases of up to five loci. Lou et al. (2003) considers an association model for quantitative traits. In contrast to the models considered here, this is a prospective model. Other measures of nonindependence for multiple markers exist such as haplotype diversity (Nei & Tajima, 1981) that becomes maximal for independent markers or mutual information (Clayton & Jones, 2012), but they do not offer a hierarchical interpretation.

We show that hierarchical LD can be seen as a reparametrization of the multinomial haplotype distribution, where every parameter corresponds to the joint cumulant of each possible subset of a set of loci (Brillinger, 1991; Thiele, 1899). Given the nature of this parametrization, this allows to directly estimate haplotype frequencies, that is without using an EM algorithm. For *M* SNPs, the parametrization consists of allele frequencies of each SNP, standard pairwise LD parameters (i.e. $D^{\prime }$), and higher order (3,…,M) LD parameters, corresponding to generalization of the pairwise LD to multiple SNPs. The proposed method is applicable to phased and unphased data and is particularly useful for detecting SNP–SNP interaction effects, long range differences in LD, the presence of “super‐alleles,” and all situations where standard haplotype analysis would be considered. We also derive bounds for the hierarchical LD parameters, which to the best of our knowledge, have not yet been provided.

In the following sections, we develop the reparametrization of the multinomial haplotype distribution, describe estimation procedures and statistical tests with reduced df for inference, and provide guidelines on how our method can be used. A simulation study, based on realistic haplotype distribution from the Wellcome Trust Case Control Consortium (WTCCC) (Burton et al., 2007) for rheumatoid arthritis (RA) and different disease generating models show that the procedure maintains the type I error rate at nominal level and has increased power over the standard single SNP or haplotype‐based association methods for a variety of realistic scenarios. We illustrate our method using unphased SNP genotype data from the data on RA and a genome wide analysis of Primary Biliary Chirrosis (Mells et al., 2011).

## 2 MATERIAL AND METHODS

### 2.1 Basic notation and assumptions

Consider the case of genotype measurements of *M* biallelic loci. Let h∈H be a haplotype at these loci, with $H={\left\{0,1\right\}}^{M}$ the set of possible haplotypes, $\left|H\right|=2^{M}$. We assume that h∼Mult(1,θ) with $\theta ={\left(\theta_{h}\right)}_{h\in H}$ the parameter vector of the haplotype frequencies, θ∈Θ and $\Theta =\{\theta |\theta \in {\left(0,1\right)}^{2^{M}},\sum_{h\in H}\theta_{h}=1\}$.

For the situation when genotypes instead of haplotypes are observed, let $G=(G_{1},\ldots ,G_{N})$ denote genotypes of *N* individuals; $D=(h_{1},h_{2})$ denotes a diplotype, that is an ordered haplotype pair, and S(g) denotes the set of diplotypes that are consistent with genotype *g*. By assuming HWE, we can model the diplotype distribution using the product distribution. Then, the likelihood of the data can be expressed as (Schaid, 2004)

$$L_{0}\left(G;\theta \right)=\prod_{i=1}^{N}\sum_{\left(h_{1},h_{2}\right)\in S\left(G_{i}\right)}\theta_{h_{1}}\times \theta_{h_{2}}.$$

In the following, we consider case‐control studies, with *N*_1_ controls, *N*_2_ cases, and sample size $N=N_{1}+N_{2}$. For genotypes $G=(G^{ca},G^{co})$ the likelihood becomes

$$\begin{array}{c} L\left(G,\theta \right)=L_{0}\left(G^{ca},\theta^{ca}\right)L_{0}\left(G^{co},\theta^{co}\right), \end{array}$$

where $\theta^{ca}$ and $\theta^{co}$ are haplotype frequencies for cases and controls, respectively. Standard haplotype testing compares haplotype frequencies of cases and controls as follows:

(1) $$H_{0}:\Theta^{0}=\left\{\left(\theta^{ca},\theta^{co}\right)\in \Theta^{2}|\theta^{ca}=\theta^{co}\right\},H_{1}:\Theta^{1}=\left\{\left(\theta^{ca},\theta^{co}\right)\in \Theta^{2}\right\}.$$

Under the null hypothesis, parameters for cases and controls are constrained to be equal, while under the alternative any parameter component can differ between the groups. The EM algorithm can be used to maximize the log‐likelihood and compute the maximum likelihood estimates under both the null and alternative hypothesis. The LR‐statistic is then

$$LR=2\left\{\log L\left(G;{\hat{\theta }}^{1}\right)-\log L\left(G;{\hat{\theta }}^{0}\right)\;\right\},$$

where ${\hat{\theta }}^{0}={\mathrm{argmax}}_{\theta \in \Theta^{0}}L\left(G;\theta \right)$ and ${\hat{\theta }}^{1}={\mathrm{argmax}}_{\theta \in \Theta^{1}}L\left(G;\theta \right)$. It follows from standard likelihood theory that LR is asymptotically $\chi_{2^{M}-1}^{2}$ distributed.

### 2.2 Reparametrization of the multinomial haplotype distribution

In order to achieve our goal of reducing the df, we present a hierarchical model of LD. To this end, Lemma 1 establishes a reparametrization δ of the multinomial haplotype frequencies θ, where every parameter corresponds to the joint cumulant of each possible subset of a set of *M* loci. We start by defining the joint cumulant.

Let $A=\{A_{1},A_{2},\ldots ,A_{M}\}$ be a set of random variables. Let $P_{A}$ refer to the set of partitions of set *A* into nonempty subsets (blocks). So, for $p\in P_{A}$, each b∈p is a block. Then, the joint cumulant of the set of random variables *A* is given as

$$\kappa \left(A\right)=\kappa \left(A_{1},A_{2},\ldots ,A_{M}\right)=\sum_{p\in P_{A}}{\left(-1\right)}^{\left|p\right|-1}\left(\left|p\right|-1\right)!\prod_{b\in p}\text{E}\left(\prod_{A\in b}A\right),$$

where |p| denotes the cardinality of set *p*.

We also use *M*‐th order cumulant to denote κ(A). The joint cumulant is a measure of how far random variables are from independence (Ahlbach, Usatine, & Pippenger, 2012). Notice that if M = 1 or M = 2, the joint cumulant reduces to the expected value and covariance, namely $\kappa \left(A_{1}\right)=\text{E}\left(A_{1}\right),\kappa \left(A_{1},A_{2}\right)=\text{E}\left(A_{1}A_{2}\right)-\text{E}\left(A_{1}\right)\text{E}\left(A_{2}\right)$.

Lemma 1 Let $A=\{A_{1},A_{2},\ldots ,A_{M}\}$ a set of *M* random variables with $A_{j}\in \left\{0,1\right\}$. For each $s\in S=2^{A}\setminus \emptyset$, let $\delta_{s}=\kappa \left(s\right)$, *that is* the joint cumulant of random variables *s*. Then $\delta ={\left(\delta_{s}\right)}_{s\in S}$ is a reparametrization of θ.

Here 2^*A*^ denotes the power set of *A*. We interpret $A_{i}$ as a biallelic locus and get that the haplotype distribution can be described by a set of cumulants for which each cumulant uniquely corresponds to a subset of the *M* loci. Note that first‐order cumulants correspond to allele frequencies and second‐ order cumulants correspond to standard pairwise LD. Thus, in cases of two SNPs, the reparametrization reduces to the standard decomposition into allele frequencies and pairwise LD parameters (Weir, 1990). A proof of Lemma 1 is given in Appendix A.1. For a set $\left\{A_{1},A_{2},A_{3}\right\}$ of random variables, we will write δ_123_ as a shorthand of $\delta_{\left\{A_{1},A_{2},A_{3}\right\}}$ and η_123_ for $\text{E}(A_{1}A_{2}A_{3})$.

As an example to illustrate the lemma, consider the case of three loci. The eight haplotype frequencies $\theta =(\theta_{000},\theta_{100},\theta_{010},\theta_{001},\theta_{110},\theta_{101},\theta_{011},\theta_{111})$ can be reparametrized into three allele frequencies, denoted by $\delta_{1},\delta_{2}$, and δ_3_, three pairwise LD parameters, denoted by $\delta_{12},\delta_{13}$, and δ_23_, and one‐third order LD parameter, denoted by δ_123_, that is $\delta =(\delta_{1},\delta_{2},\delta_{3},\delta_{12},\delta_{13},\delta_{23},\delta_{123})$. The pairwise LD parameters for all pair (j,k) of SNPs are given as

(2) $$\begin{array}{c} \delta_{jk}=\text{E}\left(A_{j}A_{k}\right)-\text{E}\left(A_{j}\right)\times \text{E}\left(A_{k}\right)=\eta_{jk}-\delta_{j}\times \delta_{k}. \end{array}$$

As in the case of pairwise LD, higher order LD parameters express the difference between observed and expected haplotype frequencies, when expected frequencies are computed under the assumption of independence, with a value of zero indicating that at least two disjoint subsets of SNPs are independent of each other, and any cumulant involving two (or more) independent SNPs will be zero (Ahlbach et al., 2012). This becomes apparent from the third‐ order LD parameter:

(3) $$\begin{array}{cc} \delta_{123} & =\eta_{123}-\delta_{1}\eta_{23}-\delta_{2}\eta_{13}-\delta_{3}\eta_{12}+2\delta_{1}\delta_{2}\delta_{3}. \end{array}$$

### 2.3 Parameter estimation

The reparametrization of the haplotype frequencies into allele frequencies and different order LD parameters introduces a hierarchy in the parameters. Specifically, higher order parameters (corresponding to singletons, pairs, triples, etc.) only depend on lower order parameters and are independent of same or higher order parameters, given the lower order ones. This hierarchical structure enables us to construct direct optimization procedures avoiding the need for an EM algorithm.

As an example, consider again the case of three SNPs. In the first step, we estimate the allele frequencies ${\hat{\delta }}_{j}$, j=1,2,3. In the second step, we estimate the pairwise LD parameters, denoted by ${\hat{\delta }}_{jk},j\neq k$, for all pairs j,k of SNPs. Notice that in (2) each $\delta_{jk}$ depends only on allele frequencies $\delta_{j}$ and $\delta_{k}$, which we have estimated in the first step, and a single parameter $\eta_{jk}$ involving a one‐dimensional optimization. Similarly, δ_123_ is estimated by a one‐dimensional optimization over η_123_ as all other terms in (3) can be recovered by applying Lemma 1 from the parameters already estimated. The whole algorithm starts with allele frequencies and performs $2^{M}-1-M$ ensuing single‐parameter optimizations.

Missing data is handled automatically by the algorithm, as missing genotypes will be excluded if and only if they are contained in a tuple for which a parameter is to be estimated. Extensions to multiple alleles per locus are straightforward but are not considered here.

### 2.4 Standardized LD parameters

LD parameters have the disadvantage of depending on allele frequencies (Hedrick, 1987). For the two locus case, Lewontin (1964) suggested normalizing the pairwise LD parameter by dividing it by achievable extremes for fixed allele frequencies:

$$\begin{array}{c} \delta_{jk}^{max}=\left\{\begin{array}{cc} \min\left(\delta_{j},\delta_{k}\right)-\delta_{j}\delta_{k}, & \;\text{if}\;\delta_{jk}\geq 0\;\text{and}\;\\ \left|\max\left(0,\delta_{j}+\delta_{k}-1\right)-\delta_{j}\delta_{k}\right|, & \;\text{if}\;\delta_{jk}<0. \end{array}\right. \end{array}$$

We suggest to generalize this concept to establish a standardized LD measure for an arbitrary number of loci. Recall that $\delta_{A}$ can be written as

$$\begin{array}{c} \delta_{A}=\eta_{A}-\sum_{p\in P_{A}\setminus A}{\left(-1\right)}^{\left|p\right|}\left(\left|p\right|-1\right)!\prod_{b\in p}\eta_{b}=\eta_{A}-\sum_{p\in P_{A}\setminus A}R_{\delta }\left(p\right), \end{array}$$

where $R_{\delta }\left(p\right)$ are terms depending on loci b∈p with |b|<M. These rest terms $R_{\delta }\left(p\right)$ are considered fixed and bounds for $\eta_{A}$ are to be determined completely analogous to the two locus case. Then

$$\delta_{A}^{\max}=\left\{\begin{array}{cc} \eta_{A}^{\max}-R_{\delta }, & \text{if}\;\delta_{A}\geq 0\;\text{and}\\ \left|\eta_{A}^{\min}-R_{\delta }\right|, & \text{if}\;\delta_{A}<0, \end{array}\right.$$

where $R_{\delta }=\sum_{p\in P_{A}\setminus A}R_{\delta }\left(p\right)$, and $\eta^{\max}$ and $\eta^{\min}$ are the upper and lower bound for $\eta_{A}$ and are defined in Appendix A.2. The standardized version of $\delta_{A}$ is then given as follows

$$\delta_{A}^{{}^{\prime }}=\frac{\delta_{A}}{\delta_{A}^{\max}}$$

A value of 1 or −1 indicates that the examined loci have not been exposed to all possible recombinations and at least one of all possible haplotype is not present in the population. $\eta_{A}^{\min}$ and $\eta_{A}^{\max}$ can be used to define the parameter space in the LD‐parametrization which we denote with Δ in the following.

### 2.5 Testing

The hierarchy present in our parametrization enables us to focus on certain orders in the hierarchy, thus sparing df as compared to testing the full distribution. We start by reformulating the global haplotype test in terms of LD parameters. Let $\delta^{ca}={\left(\delta_{s}^{ca}\right)}_{s\in S}$ and $\delta^{co}={\left(\delta_{s}^{co}\right)}_{s\in S}$ be parameter vectors for cases and controls, respectively. Then (1) can be restated as follows

(4) $$H_{0}:\Delta^{0}=\left\{\left(\delta^{ca},\delta^{co}\right)\in \Delta^{2}|\delta^{ca}=\delta^{co}\right\},H_{1}:\Delta^{1}=\left\{\left(\delta^{ca},\delta^{co}\right)\in \Delta^{2}\right\}$$

Again, $LR=2\left\{\log L\left(G;{\hat{\delta }}^{1}\right)-\log L\left(G;{\hat{\delta }}^{0}\right)\right\}\overset{D}{\to }\chi_{2^{M}-1}^{2}$ where ${\hat{\delta }}^{0}$, ${\hat{\delta }}^{1}$ are ML estimates under the null and alternative. We will refer to (4) as a *Full* test because we are testing all orders of LD parameters.

We now consider two families of tests with reduced df. The first family consists of tests that involve only lower order LD parameters. We will refer to them as *Bottom‐Up* tests (BU). Let *P* be the set containing the orders for which we would like to test for differences, for example P={1,2} if we consider both allele frequencies and pairwise LD. The corresponding null and alternative hypotheses for any such set *P* is:

$$\begin{array}{cc} H_{0}: & \Delta_{BU,P}^{0}=\left\{\left(\delta^{ca},\delta^{co}\right)\in \Delta^{2}|\forall s\in S:\left|s\right|\in P\Rightarrow \delta_{s}^{ca}=\delta_{s}^{co}\right\}\\ H_{1}: & \Delta_{BU,P}^{1}=\Delta^{1} \end{array}$$

Under *H*_0_ we only constrain parameters of orders contained in *P* to be equal.

The second family consists of tests that involve only higher order LD parameters, for example for M=3, P={2,3} focuses only on second‐ and third‐order LD parameters. We will refer to them as *Top‐Down* tests (TD). The corresponding null and alternative hypotheses for any such set *P* is:

$$\begin{array}{cc} H_{0}: & \Delta_{TD,P}^{0}=\Delta^{0}\\ H_{1}: & \Delta_{TD,P}^{1}=\left\{\left(\delta^{ca},\delta^{co}\right)\in \Delta^{2}|\forall s\in S:\left|s\right|\notin P\Rightarrow \delta_{s}^{ca}=\delta_{s}^{co}\right\} \end{array}$$

Here, parameters are constrained to be equal between cases and control both under *H*_0_ and *H*_1_ except for higher order parameters under the alternative. Both families of tests allow to employ direct optimization both under the null and the alternative. Since lower order parameters are estimated first, higher order parameters, which depend on the lower order parameters, will automatically be estimated to honor these constraints. On the other hand, had we constrained higher order parameters, lower order parameters would have to change once higher order constraints are considered. In these cases ML estimates would have to be found by joint optimization of parameters.

*Top‐Down* tests can be interpreted as performing interaction tests without correcting for main effects. Uncorrected main effect can induce apparent interactions thereby allowing to reject some hypotheses where all differences come from main effects (or orders not included). For these reasons, we will interpret these tests as global tests.

## 3 SIMULATION STUDY

To evaluate the finite sample properties of the proposed reparametrization and the association tests, we performed a simulation study. In the first part, we investigated type I error and power of the tests in data simulated based on real three‐SNP haplotype frequencies from the WTCCC RA study. Here, we focus on the four most significant associations identified from the WTCCC data analysis. In the second part, we study the performance of the tests under several disease generating models, for example SNPs with main effects only, interacting pairs of SNPs and “super‐alleles.”

In each simulated dataset, all tests described in the previous section were applied. We compare these testing strategies to a number of alternative tests. First, we test SNPs separately that corresponds to the strategy used in most genome wide analyses (*SNP* *i*). We also consider the minimum *P*‐value of these tests (*MinPvalSingle*) in order to get a combined *P*‐value. Second, we compare to an implementation of a standard haplotype analysis as implemented in R package haplo.stats (Sinnwell & Schaid, 2013). Third we compare with a method suggested by Kim, Morris, Won, & Elston (2010) that uses joint genotypes instead of haplotypes (*Kim et al*.). As implemented, this method uses pairs of adjacent SNPs and a logistic regression with a main effect for each genotype and the square of genotypes as well as an interaction term to test for association, against a null model that only contains an intercept (Table 2, Model 5 from Kim et al. (2010)). In a given SNP window, we applied the test for pairs of consecutive markers. *P*‐values for each pair are computed (Pair *i*) and a minimum *P*‐value strategy was used to combine results from all pairs in a window *MinPvalKim*. Finally, we investigated two naive strategies to evaluate the potential of sequential strategies. Method *IterHLD* is a bottom‐up strategy first testing P={1} at level α. If not rejected, the procedure tries to reject P={1,2} at level α/2 and will continue until the highest level is reached, adjusting the α level for the number of tests performed. *MinPvalHLD* selects the minimum *P*‐value of all *Bottom‐up* testing strategies.

For all simulation scenarios both under the null and alternative hypotheses, 10^3^ datasets were simulated, each consisting of 2,000 cases and 3,000 controls. Under the null hypothesis, an α‐level of 5% level is used. Under the alternative, we reject at the genome‐wide threshold of $\alpha =5\times 10^{-8}$.

### 3.1 Data simulation and results using real haplotype frequencies

For each of the four triplets identified as significant from the analysis of the WTCCC data, we estimated the haplotype frequencies in the sample of cases, the sample of controls and the pool of samples. We list these values in Table 1. The LD parameters to which these frequencies correspond are listed in Table A.1 of Appendix A.3. In order to simulate data under the null hypothesis, we draw random samples from a multinomial distribution using the frequencies estimated from the pool of samples. In order to simulate data under the alternative hypothesis, we draw random samples separately for the group of cases and controls from a multinomial distribution using the frequencies estimated in the sample of case and controls, respectively.

**Table 1:** Estimated haplotype frequencies in the cases (Ca), controls (Co). and pool (P) of cases and controls samples for each of the four triplets identified from the WTCCC data analysis (Burton et al., 2007)

	Triplet 1			Triplet 2			Triplet 3			Triplet 4		
	P	Ca	Co	P	Ca	Co	P	Ca	Co	P	Ca	Co
θ_000_	0.596	0.569	0.613	0.499	0.479	0.512	0.477	0.464	0.486	0.358	0.340	0.370
θ_001_	0.059	0.063	0.056	0.200	0.189	0.208	0.135	0.172	0.110	0.088	0.115	0.071
θ_010_	0.104	0.098	0.107	0.015	0.015	0.015	0.029	0.031	0.028	0.240	0.228	0.247
θ_011_	0.003	0.006	0.002	0.047	0.054	0.043	0.010	0.008	0.011	0.101	0.084	0.112
θ_100_	0.192	0.211	0.180	0.147	0.165	0.135	0.115	0.112	0.115	0.148	0.166	0.137
θ_101_	0.028	0.037	0.022	0.062	0.059	0.064	0.067	0.060	0.071	0.004	0.004	0.004
θ_110_	0.017	0.014	0.019	0.025	0.033	0.020	0.132	0.116	0.142	0.054	0.058	0.052
θ_111_	0.002	0.002	0.001	0.004	0.006	0.003	0.036	0.035	0.037	0.006	0.006	0.006

Results on type I error rate for all tests and triplets are listed in Table 2. At the nominal level, type I error should lie in the interval (4.68, 5.31)% for a test to properly maintain type I error. In general, the type I error rate is well maintained. With the exception of the three naive testing procedures, that is *IterHLD*, *MinPvalHLD*, and *MinPvalSingle*, all reject rates lie between 4.56% and 5.82%. Tests *MinPavlueSingle* and *MinPvalHLD* were inflated, with type I error rate around 14% and 9%, respectively. Test *IterHLD* was only slightly inflated at 6.6%.

**Table 2:** Result on type I error rate (%, α=0.05) and power (%, $\alpha =5\times 10^{-8}$) for the scenarios simulated based on parameters from significant findings from the WTCCC data

Test		df	Triplet 1	Triplet 2	Triplet 3	Triplet 4
			Type I Error Rate (%)			
*Bottom‐Up*	P={1}	3	5.13	5.31	5.21	5.06
	P={1,2}	6	4.94	4.82	4.98	4.91
*Full*		7	5.28	4.81	4.95	5.45
*MinPvalHLD*			8.90	8.86	8.83	9.13
*IterHLD*			6.70	6.79	6.63	6.55
*Top‐Down*	P={3}	1	5.27	4.91	5.08	5.33
	P={2,3}	4	5.53	5.56	5.02	5.83
Single SNP	SNP 1	1	5.05	4.89	4.83	5.12
	SNP 2	1	5.15	4.56	4.87	4.82
	SNP 3	1	5.33	5.14	5.28	4.91
*MinPvalSingle*			14.68	13.92	13.56	13.98
haplo.stats		7*	5.65	5.14	4.98	4.89
			Power (%)			
*Bottom‐Up*	P={1}	3	65.49	74.96	88.58	69.65
	P={1,2}	6	69.00	69.39	94.60	97.57
*Full*		7	65.37	65.96	97.18	97.15
*MinPvalHLD*			73.58	77.74	97.54	97.79
*IterHLD*			71.45	76.83	96.48	97.07
*Top‐Down*	P={3}	1	0.03	0.02	5.29	24.49
	P={2,3}	4	0.00	0.00	0.21	0.00
Single SNP	SNP 1	1	21.45	22.64	11.49	9.76
	SNP 2	1	0.00	17.76	1.51	10.63
	SNP 3	1	16.43	0.00	44.57	0.01
*MinPvalSingle*			33.99	35.67	51.78	19.06
haplo.stats		7*	68.43	69.12	97.28	97.19

Parameter values for each scenario are listed in Table A.1

*df might be different because the package automatically groups rare haplotypes.

The power for all tests and triplets is also listed in Table 2. In all triplets, the single SNP test, the *MinPvalSingle* and both *Top‐Down* tests reach power below 70%. Regarding the other tests, different tests seem to be more powerful in each triplet with the *IterHLD* test and *Bottom‐Up* test for P={1,2} being the ones with the most consistent power across all triplets. In all triplets the score test from haplo.stats performs comparable to the *Full* test or the *Bottom‐Up* test for P={1,2}.

### 3.2 Data simulation and results under different disease generating models

In this section, we further study the type I error rate and power properties of each test under different disease models and different LD structures. In all scenarios, we considered four SNPs with allele frequencies equal to .05, .18, .31, and .45, respectively. Two structures of LD among the SNPs are considered. In Scenario 1, the SNPs were in equilibrium, thus all second, third and fourth LD parameters were equal to zero. In Scenario 2, the second‐order standardized LD parameters were set to .4, the third‐order LD standardized parameters were set to .1 and the fourth‐order LD parameter was set to zero. In both cases, we mapped the LD parameters to haplotype frequencies, which are listed in Table A.2 of Appendix A.3, and used those frequencies to generate haplotype data for a large population of individuals. The LD parameters in Scenario 1 correspond to frequencies in which 11 out of 16 haplotypes had frequencies below 5% and six had frequencies below 1%. On the other hand, in Scenario 2 only four haplotypes had frequencies below 5%.

Using different disease models, we generate the disease status *Y* of each individual and then sampled 2,000 individual from the population of cases and 3,000 individuals from the population of controls. For each disease model the following logistic model was used

(5) $$\text{logit}\left\{\text{P}(Y=1|D)\right\}=\alpha_{0}+\sum_{j=1}^{4}\alpha_{j}G_{j}+\sum_{j,k=1,j\neq k}^{4}\alpha_{ij}G_{j}\times G_{k}+\sum_{s\in S}\gamma_{s}SA_{s},$$

where α_0_ is the intercept; $\alpha_{j},j=1,\ldots ,4$ are the main effect odds ratios of each SNP, $\alpha_{jk},j,k=1,\ldots ,4,j\neq k$ are the interaction effect for each pair of SNP; $\gamma_{s}$ are the main effects of the “super‐allele” at loci s∈S, with S={{2,3},{1,2,3},{1,2,3,4}} and

$$\begin{array}{c} SA_{23}=\left\{\begin{array}{cc} 0 & \;\text{if}\;\text{both}\;h_{1}\;\text{and}\;h_{2}\notin D_{23}\\ 1 & \;\text{if}\;\text{one}\;\text{of}\;h_{1},h_{2}\in D_{23}\\ 2 & \;\text{if}\;\text{both}\;h_{1}\;\text{and}\;h_{2}\in D_{23} \end{array}\right.,\;SA_{123}=\left\{\begin{array}{cc} 0 & \;\text{if}\;\text{both}\;h_{1}\;\text{and}\;h_{2}\notin D_{123}\\ 1 & \;\text{if}\;\text{one}\;\text{of}\;h_{1},h_{2}\in D_{123}\\ 2 & \;\text{if}\;\text{both}\;h_{1}\;\text{and}\;h_{2}\in D_{123} \end{array}\right., \end{array}$$

$$\begin{array}{c} SA_{1234}=\left\{\begin{array}{cc} 0 & \;\text{if}\;h_{1}\neq \text{“1111”}\;\text{and}\;h_{2}\neq \text{“1111”}\\ 1 & \;\text{if}\;h_{1}=\text{“1111”},h_{2}\neq \text{“1111”}\;\text{or}\;h_{1}\neq \text{“1111”},h_{2}=\text{“1111”}\\ 2 & \;\text{if}\;h_{1}=\text{“1111”}\;\text{and}\;h_{2}=\text{“1111”} \end{array}\right., \end{array}$$

and $D_{23}=\left\{\text{“0110”},\text{“1110”},\text{“0111”},\text{“1111”}\right\}$, that is all haplotypes that contain the “1” allele at loci 2 and 3 and $D_{123}=\left\{\text{“1110”},\text{“1111”}\right\}$ the haplotypes that contain the “1” allele at loci 1, 2, and 3.

Under the null hypothesis, all parameters in (5), besides the intercept, were zero. Results on type I error rate for all tests and scenarios are listed in Table 3. Rejection rates for the three *Bottom‐Up* tests, the *Full* test, the three *Top‐Down* tests, the four single SNP tests and the two *Kim et al*. tests lie between 4.13% and 5.78%. Type I error rate for haplo.stats is 6.55% and 4.67%. Inflation of *IterHLD* is slightly higher still at 7%. *MinPvalHLD*, *MinPavlSingle*, and *MinPvalKim* show strong inflation, rejection rates of 10%, 18%, and 10%, respectively.

**Table 3:** Result on type I error rate (%, α=0.05) for scenarios simulated under different disease generating models

			Type I Error Rate	
Test		df	Scenario 1	Scenario 2
*Bottom‐Up*	P={1}	4	5.15	5.27
	P={1,2}	10	5.14	5.24
	P={1,2,3}	14	4.78	4.44
*Full*		15	4.59	4.31
*IterHLD*			7.03	7.14
*MinPvalHLD*			10.39	10.22
*Top‐Down* Tests	P={4}	1	4.54	5.78
	P={3,4}	5	4.35	4.64
	P={2,3,4}	11	4.45	4.13
Single SNP	SNP 1	1	4.82	5.21
	SNP 2	1	5.05	5.37
	SNP 3	1	4.93	4.91
	SNP 4	1	4.90	5.03
MinPvalSingle			18.38	18.47
haplo.stats		15*	6.55	4.68
*Kim et al*	Pair 1	5	5.29	5.72
	Pair 2	5	5.01	4.78
*MinPvalKim*			10.05	10.20

Parameter values for each scenario are listed in Table A.2

* df might be different because the package automatically groups rare haplotypes.

For scenarios under the alternative hypothesis, six different disease models were considered. In Model 1, the four SNPs had only main effects on disease risk. In Model 2, SNP 2 and 3 had main and interaction effects on disease risk. In Model 3, SNPs 1, 2, and 3 had only interaction effects. We also studied the power of our approach in the presence of “super‐alleles.” In this case, we assumed that the combination of alleles over two, three, and four SNPs also had an effect of disease risk. In Model 4, SNP 2 and 3 and the haplotype “11” over these two loci had a main effect; in Model 5, SNP 1, 2, and 3 and the haplotype “111” had a main effect and in Model 6, all four SNPs and the haplotype “1111” had a main effect. Results on power for all tests and models, as well as the exact parameter values for each model, are listed in Table 4 for Scenario 1 and in Table 5 for Scenario 2.

**Table 4:** Power results for Scenario 1 (%, $\alpha =5\times 10^{-8}$)

			Power					
Test		df	Model 1	Model 2	Model 3	Model 4	Model 5	Model 6
*Bottom‐Up*	P={1}	4	90.24	90.84	69.78	65.94	29.53	13.83
	P={1,2}	10	73.79	85.76	88.70	62.80	20.19	10.97
	P={1,2,3}	14	63.16	77.91	81.83	51.59	14.95	9.74
*Full*		15	60.76	75.91	80.26	48.94	13.90	9.08
*IterHLD*			90.29	91.86	87.75	70.22	31.21	16.45
*MinPvalHLD*			90.37	92.27	89.61	71.85	32.15	17.84
*Top‐Down* Tests	P={4}	1	0.00	0.00	0.00	0.00	0.00	0.00
	P={3,4}	5	0.00	0.00	0.00	0.00	0.00	0.00
	P={2,3,4}	11	0.00	0.00	1.66	0.00	0.00	0.01
Single SNP	SNP 1	1	0.03	0.00	4.43	0.00	0.00	0.10
	SNP 2	1	3.04	79.20	17.49	30.50	1.94	0.14
	SNP 3	1	10.74	11.10	3.21	15.10	1.30	0.25
	SNP 4	1	15.57	0.00	0.00	0.00	0.90	0.34
MinPvalSingle			27.04	81.25	23.53	40.65	4.09	0.83
haplo.stats		15*	64.21	78.82	81.58	54.40	18.17	22.29
*Kim et al*	Pair 1	5	2.10	53.55	46.70	11.35	0.42	0.85
	Pair 2	5	47.56	2.74	0.47	4.01	5.49	0.89
*MinPvalKim*			48.67	54.78	46.93	14.86	5.89	1.72

Nonzero parameters for Model 1: $\alpha_{i}=\log\left(1.2\right)$, i=1,…,4; Model 2: $\alpha_{2}=log\left(1.2\right),\alpha_{3}=log\left(1.1\right)$, $\alpha_{23}=log\left(1.2\right)$; Model 3: $\alpha_{jk}=log\left(1.3\right)$, j,k=1,2,3, j≠k; Model 4: $\alpha_{2}=\alpha_{3}=log\left(1.1\right)$, $\gamma_{23}=log\left(1.5\right)$; Model 5: $\alpha_{i}=log\left(1.1\right),k=2,3,4$, $\gamma_{234}=log\left(1.5\right)$; Model 6: $\alpha_{i}=log\left(1.1\right)$, i=1,2,3,4, $\gamma_{1234}=log\left(5\right)$

*df might be different because the package automatically groups rare haplotypes.

**Table 5:** Power results of each test on Scenario 2 (%, $\alpha =5\times 10^{-8}$)

			Power					
Test		df	Model 1	Model 2	Model 3	Model 4	Model 5	Model 6
*Bottom‐Up*	P={1}	4	72.95	94.70	81.76	78.77	86.70	79.31
	P={1,2}	10	47.81	89.14	80.27	65.76	74.49	71.28
	P={1,2,3}	14	35.48	81.36	69.80	52.33	64.64	62.47
*Full*		15	33.19	79.69	68.04	50.33	62.67	60.75
*IterHLD*			73.00	95.12	85.06	79.64	87.31	81.42
*MinPvalHLD*			73.10	95.24	85.93	80.16	87.67	82.20
*Top‐Down* Tests	P={4}	1	0.00	0.00	0.00	0.00	0.00	0.00
	P={3,4}	5	0.00	0.00	0.00	0.00	0.00	0.00
	P={2,3,4}	11	0.00	0.01	0.01	0.00	0.00	0.00
Single SNP	SNP 1	1	1.61	0.00	31.24	0.01	0.00	17.36
	SNP 2	1	38.07	86.59	49.42	66.91	51.38	16.20
	SNP 3	1	10.72	66.42	11.92	38.04	40.71	15.93
	SNP 4	1	6.93	0.11	0.00	0.00	23.63	10.14
MinPvalSingle			45.84	92.79	64.03	75.17	70.60	42.64
haplo.stats		15*	36.84	80.80	72.23	54.34	65.60	66.56
*Kim et al*	Pair 1	5	30.97	65.51	74.01	38.73	25.93	37.72
	Pair 2	5	13.82	39.86	4.60	15.72	50.10	20.96
*MinPvalKim*			38.24	74.62	74.43	45.44	58.92	47.57

Nonzero parameters for Model 1: $\alpha_{1}=\alpha_{2}=log\left(1.2\right)$, $\alpha_{3}=\alpha_{4}=log\left(1.1\right)$ ; Model 2: $\alpha_{2}=\alpha_{3}=log\left(1.1\right)$, $\alpha_{12}=log\left(1.2\right)$; Model 3: $\alpha_{jk}=log\left(1.2\right)$, j,k=1,2,3, j≠k; Model 4: $\alpha_{1}=\alpha_{2}=log\left(1.1\right)$, $\gamma_{23}=log\left(1.3\right)$; Model 5: $\alpha_{i}=log\left(1.1\right),k=1,2,3$, $\gamma_{123}=log\left(1.3\right)$; Model 6: $\alpha_{i}=log\left(1.1\right)$, i=1,2,3,4, $\gamma_{1234}=log\left(2\right)$

*df might be different because the package automatically groups rare haplotypes.

Based on these results, we make the following observations. First, as expected, in the presence only of main effects, that is Model 1, for both Scenarios (1:, 2:), the most powerful test is the *Bottom‐Up* test with P={1} (1: 90%, 2: 73%). Second, although the *Bottom‐Up* test with P={1} does not include second‐order parameters, its power is comparable to the power of *Bottom‐Up* test with P={1,2} in the presence of both main and interaction effects, that is Model 2 (1: 91% *v*s 86%, 2: 95% *v*s 91%), or in the presence only of interacting effects, that is Model 3 (1: 70% *v*s 89%, 2: 81% *v*s 80%). In the presence of “super‐alleles,” the power to detect association when the LD among the involved loci is zero and the effect is spread across three or four loci, that is Model 5 (< ≈ 30% for all tests) and 6 (< ≈ 20% for all tests) in Scenario 1, is much lower compared to the power in the presence of LD, that is Models 5 and 6 in Scenario 2 (> ≈ 30% for some *Buttom‐Up* models, *IterHLD, MinPvalHLD*). For both scenarios and all models, except Model 6 for Scenario 1, at least one of the *Bottom‐Up* tests is more powerful than haplo.stats. The *Bottom‐Up* test with P={1,2} was the one with the most consistent power across all models and scenarios (>70% in 8 out of 12 cases). Most importantly, test *IterHLD* shows very consistent results. In view of the slight inflation of this test (type I error ≈7%), “true” power would be slightly lower, but the consistency across scenarios suggest that the strategy underlying *IterHLD* is very promising. The *Top‐Down* tests show essentially power of zero. When evaluated at the 5% level, power is ≈5% in 20 out of the 32 models and is >80% only in two cases. This is discussed below.

## 4 DATA EXAMPLE

### 4.1 Candidate loci analysis

To illustrate an application of the proposed association tests, we performed an analysis of a dataset from the WTCCC, consisting of 1,860 cases of RA and 2,938 controls. In the initial analysis, single SNP tests were performed and several SNPs, strongly associated with RA, were identified (Burton et al., 2007). In addition, a list of 59 SNPs, showing “moderate” association with RA, with nominal significance in the range of 10^−3^ to 10^−6^, was provided in the initial article. Some of these SNPs map to genes with plausible biological relevance however the single SNP analyses failed to pass the significance threshold.

Here, we investigate possible increase in the significance level of the 59 SNPs when a three SNP haplotype‐based analysis is used. For each of these SNPs we choose 40 neighboring SNPs that had passed quality control, 20 to the left and 20 to the right side of the SNP and construct all possible triplets between the SNPs that contain the moderately associated SNP. For each of the 59 SNP, 780 triplets were constructed. To avoid problems caused by high LD, we excluded from the analysis all triplets in which at least one of the standardized pairwise LD parameters was above 0.8. For the remaining triplets, the tests mentioned in the previous section were applied. A triplet of SNPs was considered to be associated with RA if the *P*‐value was below the threshold $5\times 10^{-8}/\left(N_{tests}\times N_{triplets}\right)$, where $N_{tests}=5$ is the total number of tests performed on each triplet and $N_{triplets}$ the total number of triplets tested for each “moderately” associated SNP. We also applied the *IterHLD* strategy with $\alpha =5\times 10^{-8}/N_{triplets}$. For comparison purposes, we also show results from the single‐SNP analysis.

Several triplets containing the SNPs rs12723859 and rs12205634 showed a strong association with RA. Specifically, for rs12723859 we identified 40 triplets with 20 unique SNPs, and for rs12205634 we identified five triplets with four unique SNPs (see Supplementary Information). For rs6920220, three triplets consisting of four unique SNPs, had *P*‐values smaller than the genome‐wide significance threshold $5\times 10^{-8}$ but they were no longer significant when adjusting for the multiple number of tests and triplets. For the other 56 SNPs, no strong association with RA was identified from the haplotype analysis. In Table 6, we list for each of rs6920220, rs12723859, and rs12205634, the *P*‐values of all tests for the two triplets that show the strongest association with RA. For rs6920220, we tested a total of 21 triplets. Only the *Bottom‐Up* test for allele frequencies yields a *P*‐value below $5\times 10^{-8}$. If we correct for the number of tests and triplets tested no test yields a significant *P*‐value. For rs12723859 and rs12205634, we tested a total of 144 and 38 triplets, respectively. The *Full* test and the *Bottom‐Up* tests for P={1} and P={1,2} yield *P*‐values below $5\times 10^{-8}$. After correcting for the number of tests performed the *Bottom‐Up* tests for P={1} no longer gives a significant association, the *Bottom‐Up* tests for P={1,2} is still significant.

**Table 6:** Results on real data

SNPs in the triplet		*Bottom‐Up* Test		*Full* Test	*Top‐Down* Test		Single SNP tests		
		P={1}	P={1,2}		P={3}	P={2,3}			
SNP rs6920220									
rs11961920	rs11970411	2.6e‐08	7.9e‐08	2.3e‐07	0.89	0.21	5e‐06	0.16	1.2e‐05
rs11970411	rs674451	8.5e‐09	9.9e‐08	2.8e‐07	0.81	0.56	5e‐06	1.2e‐05	0.25
SNP rs12723859									
rs12739961	rs1113523	1.8e‐10	4.4e‐11	5.6e‐12	7.78e‐03	8.50e‐04	3e‐05	0.0013	2.2e‐07
rs12739961	rs17013326	2.4e‐10	7.3e‐11	7.9e‐12	6.40e‐03	9.29e‐04	3e‐05	0.0013	3.1e‐07
SNP rs12205634									
rs411136	rs210137	1.9e‐08	4.8e‐12	1.2e‐11	0.41	2.26e‐05	5.2e‐05	4.3e‐05	6.9e‐02
rs411136	rs210138	2.1e‐08	1.1e‐11	2.1e‐11	3.7e‐05	5.1e‐05	5.2e‐05	4.3e‐05	6.9e‐02

Using the *IterHLD* strategy, we find 57 significant triplets for rs12723859, 39 of which are rejected at the P={1} level, 17 are rejected at the P={1,2} level, and one which is only rejected at the full level (see Supplementary Information). Similarly, for rs12205634, we find the same four triplets described above, all of which are rejected at the P={1,2} level. We do not find any significant triplets for rs6920220 after adjusting for multiple testing.

### 4.2 Genome‐wide analysis

In addition to the analysis of candidates from the RA dataset, we also analyzed a full genome wide dataset for which we acquired a case‐control dataset on Primary Biliary Chirrosis from the European Genome‐Phenome Archive (EGAS00000000039). Our study was approved by the data access committee of the WTCCC3 datasets. Details on the dataset are given elsewhere (Mells et al., 2011). We excluded markers and individuals as provided from the original study. According to the study protocol, variants with an exact *P*‐value below 10^−6^ for HWE were excluded from the analysis. Additionally, we filtered markers at a minor allele frequency of 0.15 and pruned LD using *plink* (Purcell et al., 2007) (window size 50, shift 5, VIF 2) for moderate LD to avoid collinearity problems. After these selections, 97442 SNPs remained in the dataset that were analyzed marginally using logistic regression. Haplotype tests for pairs and triples were employed in a sliding window approach. The data contained 1,906 cases and 2,859 controls after quality control.

**Figure 1:**
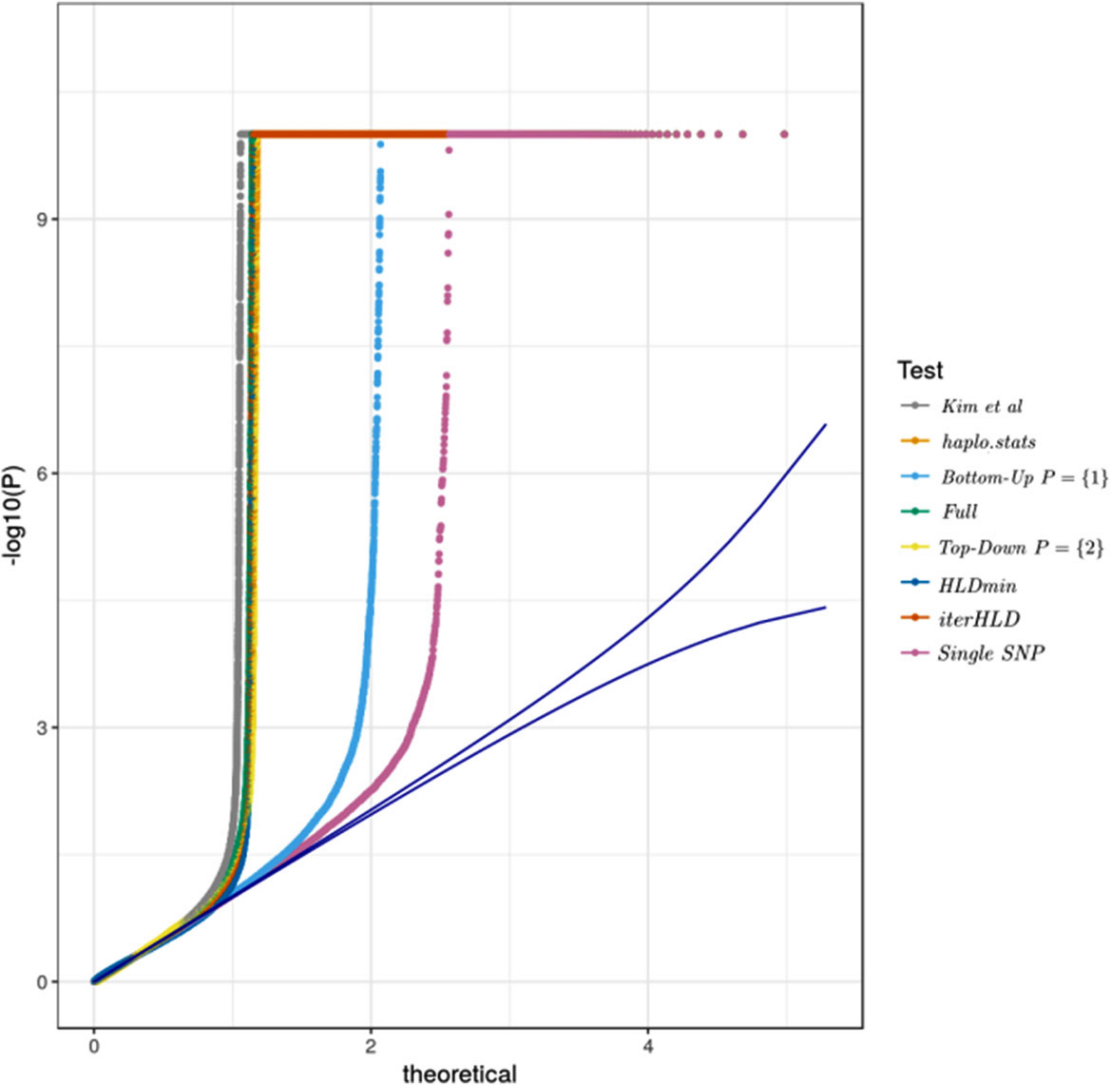
QQ plot for all two locus tests, including the marginal test. *P*‐values were inflation‐corrected before plotting (see text). Blue lines represent point‐wise confidence limits for ordered *P*‐values

**Figure 2:**
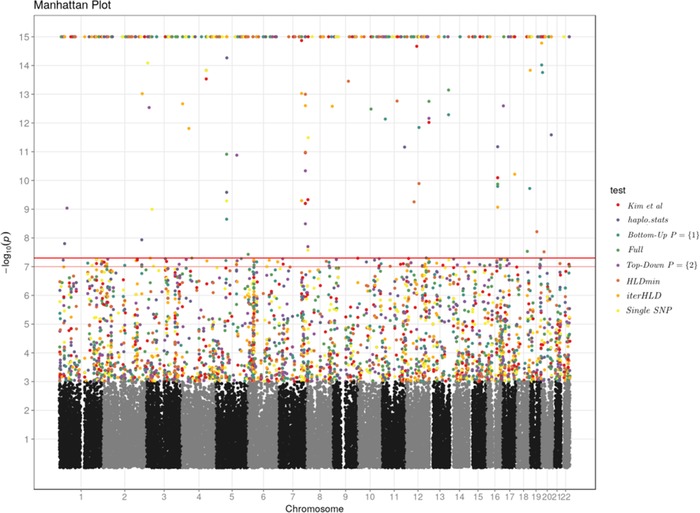
Manhattan plot of *P*‐values for all test when haplotypes span **two** loci. Colors are applied to *P*‐values $<10^{-3}$. *P*‐values $<5\times 10^{-8}$ were filtered by the *tower criterion* (see text). *P*‐values $<10^{-15}$ were truncated

Inflation factors (IFs) (Devlin & Roeder, 1999; Yang et al., 2011) for the tests are shown in Table 7. The original publication reports an IF of 1.09. The marginal baseline model using logistic regression had an IF of 1.06 indicating moderate inflation. All models using two loci had higher IFs with the model *Bottom‐Up*.1 performing best in terms of inflation with an IF of 1.18. As expected, *haplo.stats*, *Bottom‐Up‐Full* had identical IFs of 1.36. QQ‐plots for the tests are shown in Figure 1. Analyzing three loci simultaneously, IFs increased for the models, with the exception of *Bottom‐Up* with P={1,2}, *Bottom‐Up‐Full*, and *Top‐Down* with P={2,3}. In the latter cases, an increase in inflation was masked by a peak of *P*‐values in the histogram close to 1, indicating numeric problems when performing the model fit. Owing to the inflation of results, all findings have to be interpreted strictly exploratorily. To this end, we have limited locations shown in the Manhattan plots to those that had a local correlation between log‐*P*‐values and position. The precise definition of this filtering step is given in the supplement (*Tower criterion*). The number of hits with this local support are shown in Table 7. Manhattan plots for pairs of SNPs are shown in Figure 2. A list of loci for which at least three tests agreed at a threshold of $5\times 10^{-8}$ and had local support is shown in Table 8.

**Table 7:** Summary of GWAS analyses by all test for haplotypes spanning 2 or 3 loci (number after ':')

Test:2	Infl.:2	#:2	#T:2	Test:3	Infl.:3	#:3	#T:3
*Single SNP*	1.06	277	22	–	–	–	–
*Kim et al*	1.43	8690	33	–	–	–	–
*haplo.stats*	1.37	6836	84	*haplo.stats*	1.53	10824	230
*Bottom‐Up* P={1}	1.18	889	59	*Bottom‐Up* P={1}	1.38	1047	26
–	–	–	–	*Bottom‐Up* P={1,2}	1.27	4201	35
*Full*	1.36	7151	17	*Full*	1.29	4247	29
–	–	–	–	*Top‐Down* P={2,3}	1.17	3945	33
*Top‐Down* P={2}	1.20	6662	26	*Top‐Down* P={3}	1.40	3465	341
*HLDmin*	1.97	7156	18	*HLDmin*	2.66	4613	22
*iterHLD*	1.57	7145	18	*iterHLD*	1.98	4608	24

*Test* is the name of the test. *Infl*. denotes the inflation factor, # denotes the number p‐values $<5\times 10^{-8}$. #T denotes number of significant *P*‐values filtered by the tower criterion (see text). *Single SNP* is given for reference and refers to a SNP‐by‐SNP logistic regression

**Table 8:** Positions (*chr, pos*) and *P*‐values for tests for which at least three tests reached a *P*‐value $<5\times 10^{-8}$ when haplotypes span two loci

chr	pos	Kim et al	Full	HLDmin	iterHLD	haplo.stats	*Top‐Down* P={2}	*Bottom‐Up* P={1}	Singe SNP
1	31008523	1.8e‐249	3.3e‐309	3.3e‐309	6.5e‐309	8.6e‐171	7.1e‐311	–	–
1	31032093	1.7e‐28	2.9e‐33	2.9e‐33	5.8e‐33	–	7.4e‐35	–	–
1	31033624	1.5e‐185	1.1e‐224	1.1e‐224	2.2e‐224	1.6e‐08	2.7e‐226	–	–
1	90440693	1.8e‐32	–	1.2e‐33	2.3e‐33	6.0e‐33	–	–	–
1	90481133	9.6e‐185	–	2.1e‐224	4.2e‐224	5.4e‐165	–	–	–
5	59580410	2.2e‐09	2.6e‐10	2.6e‐10	5.2e‐10	–	–	–	–
5	122166044	–	–	4.1e‐151	8.2e‐151	2.1e‐119	–	–	–
5	153251949	–	3.3e‐189	3.3e‐189	6.5e‐189	2.3e‐140	–	–	–
6	26564048	1.7e‐251	5.5e‐302	5.5e‐302	1.1e‐301	5.5e‐172	–	–	–
7	88709037	–	3.4e‐140	–	–	6.7e‐145	4.5e‐90	–	–
7	149498823	2.9e‐100	3.2e‐09	6.3e‐10	6.3e‐10	1.0e‐11	–	6.3e‐10	–
7	149504002	–	4.6e‐11	1.1e‐11	1.1e‐11	1.0e‐13	–	1.1e‐11	2.5e‐13
12	68141467	–	8.8e‐79	8.8e‐79	1.8e‐78	1.1e‐68	–	–	–
12	99342932	5.1e‐56	1.0e‐59	1.0e‐59	2.0e‐59	1.2e‐54	–	–	–
13	82025812	8.4e‐246	1.1e‐320	1.1e‐320	2.1e‐320	–	1.5e‐323	–	–
16	60188415	8.5e‐10	8.0e‐11	8.0e‐11	1.6e‐10	1.3e‐10	–	–	–
17	66951514	–	1.1e‐35	1.1e‐35	2.2e‐35	–	–	–	–
18	56526010	–	2.5e‐162	2.5e‐162	5.0e‐162	3.3e‐126	9.9e‐165	–	–
19	63603580	4.5e‐17	6.6e‐19	6.6e‐19	1.3e‐18	3.1e‐18	8.9e‐21	–	–

*P*‐values were filtered by the *tower criterion* (see text)

## 5 DISCUSSION

In this article, we propose a reparametrization of the multinomial haplotype distribution into allele frequencies, standard pairwise LD parameters, and higher order LD parameters. Our reparametrization enables us to employ flexible testing strategies over a range of parameter sets. For example, joint tests of single‐SNPs and joint tests of single‐SNPs and their pair‐wise LD. We showed in both simulated and real data that such tests can often have increased power as compared to the full global haplotype or single‐SNP based tests.

In this study, we use rather simplistic multiple testing strategies, namely using a Bonferroni correction for multiple tests performed on the same genotype data. This is certainly not optimal as the performed tests are usually highly correlated. Among our future interests is to develop iterative or sequential testing procedures, for example (Meinshausen, 2008), which better exhaust the α level. Another option is to use information criteria such as AIC or BIC for model selection. Moreover, we have not focused on the choice of haplotype size or region covered as an optimal strategy. It is likely that the optimal number of SNPs used for haplotype‐based approaches will depend on the population history and the genomic region, which is beyond the scope of this report. We are currently working on implementation of the hierarchical LD model in the context of equivalence testing for reconstruction of independent haplotype blocks, which, apart from gains in statistical efficiency, would help to obviate SNP pruning in genome wide datasets.

Even with this conservative strategy, we could demonstrate a new association that makes our method an interesting alternative for the analysis of genome wide data. The exploratory investigation of the methods *IterHLD* and *MinPvalHLD* indicate that power gains are to be expected by better exhaustion of the α level as compared to a simple Bonferroni correction. Especially *IterHLD* seems to offer a worthwhile testing strategy. Proper α‐level control would need to be developed in future work. We stress that the data analysis should be considered exemplary and more general conclusions require more extensive data analyses. In our simulations *Bottom‐Up* performed better than *Top‐Down* procedures. The estimation of higher order parameters depends on lower order parameters. This implies reduced precision of estimates when going up the hierarchy and explains the findings for *Top‐Down* procedures. This can be exploited by testing accurately estimated parameters first as done by *IterHLD* and is another component in the power trade‐off in the HLD framework apart from reducing df.

For a case‐control sample, population substructure and cryptic relatedness among subjects lead to overdispersion of the chi‐square test statistic for association and causes spurious rejections of the null hypothesis. The dataset we are using for the candidate gene analysis is known to be fairly homogeneous (Burton et al., 2007) and we did not expect population stratification artifacts. In this analysis, we found only small differences in haplotype frequencies (⩽6%) between cases and controls, but nevertheless suggest these could be relevant. Our GWAS analysis on the other hand shows that haplotype‐based analyses are indeed very sensitive to population stratification as indicated by IFs. Careful control of inflation seems necessary. As presented, our method does not allow incorporation of additional covariates. An option to deal with covariates at the moment is to perform stratified analyses in a Mantel–Haenszel framework. Due to the excessive anti‐conservative nature of all tests in the GWAS analysis, we do not discuss specific loci. When comparing the testing options, we note, that our family of tests has the advantage over the competing methods considered here that the complexity in terms of degrees of freedom and parameter interpretation can be controlled. For example, the *Bottom‐Up* procedure (P={1}) had a clearly lower IF as compared to the other tests. A limitation of our GWAS analysis is the stringent LD pruning employed that was necessary to run all tests on all SNP sets. Results presented in Tables 7 and 8 are expected to change with different pruning criteria, however, the ability to control robustness by chosing dfs should be unaffected.

To avoid diminished power from the large number of haplotype configurations, Schaid et al. (2002) proposed to either pool rare haplotypes into a single baseline group or to scan a large chromosomal region for subsegments that may be associated with the trait, starting with single‐locus associations, followed by “sliding” tests for two‐locus haplotypes, followed by “sliding” tests for three‐locus haplotypes, and so forth. We saw from our simulation study that, as the number of haplotype configuration increases, pooling rare haplotypes does not avoid the diminished power problem. In addition, analyses involving a series of adjacent markers assume that the most informative markers are the physically closest. However, this is not always the case and tests based on such associations will not always be optimal. Consider for example the case when relatively recent mutations have introduced correlation among two SNPs in a low LD region, with for example five SNPs separating them. In order to include the pairwise correlation of the two SNPs of interest, we would have to use a sliding window of size 7 and perform a test with $2^{7}-1=127$ df. Given the large number of haplotype configurations, most haplotype frequencies will be very low and pooling most haplotypes would be unavoidable. On the other hand, one could repeat the same procedure, using again a sliding window of 7, but testing only for allele frequencies and pairwise LD parameters. In this case, one would need to perform a test with 7+72=28 df. In this study, we followed a similar, heuristic strategy that lead to the identification of novel associations. Regarding computational efficiency, we offer a way to estimate haplotype frequencies directly, that is without an EM algorithm. Conceptually, this should lead to improved performance although we did not investigate this systematically. For larger number of loci (say >7), we expect that that heuristic strategies to limit the number of parameters will dominate run‐time performance rather than choice of parametrization.

In a given population, the mutations that are causal in disease etiology will have arisen on one or more ancestral haplotypes (Degli‐Esposti, Leelayuwat, & Dawkins, 1992) and thereafter will have spread to other haplotypes by recombination. Early on in this process, very‐high‐order association will exist, and the most powerful test for association will be a very‐high‐order association test, since the strength of the high‐order effect more than outweighs the large number of df. However, this advantage will not survive in perpetuity, since, as shown in Clayton and Jones (1999) high‐order effects will be rapidly diluted by recombination, at progressively more rapid rates than first‐order association between a single marker or a pair of markers and disease. As a result, tests based on lower order effects will in general be more powerful than the full haplotype tests. This result is also supported by our simulation study, since in the scenarios we considered, *Bottom Up* tests are the most powerful across all different disease models. Our proposed method allows to flexibly accommodate both higher and lower order LD scenarios. The test for joint marginal effects seems to be particularly relevant for many situations.

## CONFLICT OF INTEREST

The authors have declared no conflict of interest.

## Supporting information

Supporting Information

Supporting Information

## APPENDIX 1

### A.1 Proof of lemma 1

Consider again the case of genotype measurements of *M* biallelic loci. Let h∈H be a haplotype at these loci, with $H={\left\{0,1\right\}}^{M}$ enumerating the 2^*M*^ possible haplotypes. Assume that h∼Mult(1,θ) with $\theta ={\left(\theta_{h}\right)}_{h\in H}$ the parameter vector of the haplotype frequencies of each haplotype, θ∈Θ and $\Theta =\{\theta |\theta \in {\left(0,1\right)}^{2^{M}},\sum_{h\in H}\theta_{h}=1\}$. Let $A=\{A_{1},A_{2},\ldots ,A_{M}\}$ a set of *M* random variables with $A_{j}\in \left\{0,1\right\}$ the indicator random variable for either one of the two alleles at locus j,j=1,…,M. Let $S=2^{A}\setminus \emptyset$ be the power set of *A*, in lexicographical order, without the empty set, that is, the set of all singletons, pairs, triplets, etc. of allele indicator random variables.

In order to prove that δ is a reparametrization of θ, we introduce an intermediate parametrization, denoted as η. Then the proof goes as follows. First, we show that η is a reparametrization of θ. To prove this, we introduce the function f(θ,S) and prove that *f* is bijective. Then, we show that δ is a reparametrization of η, which implies that δ is also a reparametrization of θ. Similarly, to prove this, we introduce the function q(η,S) and prove that *q* is bijective.

#### Mapping function g

For a set of random variables s∈S, let $\tau_{s}=\left\{v\in {\left\{0,1\right\}}^{M}|A_{j}\in s\Leftrightarrow v\left[j\right]=1\right\}$ a tuple of all haplotypes whose *j*‐th element is 1 if $A_{j}\in s$. We define *g* to be a function that takes as an input the parameter vector $\theta ={\left(\theta_{h}\right)}_{h\in H}$ and outputs the joint expectation of random variables in *s*, which we denote with $\eta_{s}$. That is,

$$\begin{array}{c} g\left(\theta ,s\right)=E\left(\prod_{A\in s}A\right)=\sum_{h\in \tau_{s}}\theta_{h}=\eta_{s}. \end{array}$$

We illustrate *g* with the following example. Let M=3 and $s=\{A_{1},A_{2}\}$. Then $\tau_{s}$ will contain two haplotypes, that is $\tau_{\left\{A_{1},A_{2}\right\}}=\left\{\left(1,1,0\right),\left(1,1,1\right)\right\}$, and $g\left(\theta ,\left\{A_{1},A_{2}\right\}\right)=E\left(A_{1}A_{2}\right)=\theta_{\left(1,1,0\right)}+\theta_{\left(1,1,1\right)}$. We are thus computing the joint expectation of *A*_1_ and *A*_2_ or the haplotype frequency for loci 1 and 2 with allele 1 chosen at each locus.

For a haplotype (1,1,1), we will write θ_111_ as a shorthand of θ_(1, 1, 1)_. Similarly, for a set $\left\{A_{1},A_{2},A_{3}\right\}$ of random variables, we will write η_123_ as a shorthand of $\eta_{\left\{A_{1},A_{2},A_{3}\right\}}$.

#### Mapping function f

We define *f* to be a function that takes as input the parameter vector $\theta ={\left(\theta_{h}\right)}_{h\in H}$ and outputs the joint expectation of random variables in *s* for all s∈S. That is,

$$\begin{array}{c} f\left(\theta ,S\right)={\left\{g\left(\theta_{\tau_{s}},s\right)\right\}}_{s\in S}={\left(\sum_{h\in \tau_{s}}\theta_{h}\right)}_{s\in S}={\left(\eta_{s}\right)}_{s\in S}. \end{array}$$

We illustrate *f* with the following example. For M=3 markers,

$$\begin{array}{c} S=\left\{\left\{A_{1}\right\},\left\{A_{2}\right\},\left\{A_{3}\right\},\left\{A_{1},A_{2}\right\},\left\{A_{1},A_{3}\right\},\left\{A_{2},A_{3}\right\},\left\{A_{1},A_{2},A_{3}\right\}\right\}. \end{array}$$

Moreover $\tau_{A_{1}}=\left\{\left(1,1,1\right),\left(1,1,0\right),\left(1,0,1\right),\left(1,0,0\right)\right\}$, $\tau_{A_{2}}=\left\{\left(1,1,1\right),\left(1,1,0\right),\left(0,1,1\right),\left(0,1,0\right)\right\}$, $\tau_{A_{3}}=\left\{\left(1,1,1\right),\left(0,1,1\right),\left(1,0,1\right),\left(0,0,1\right)\right\}$, $\tau_{\left\{A_{1},A_{2}\right\}}=\left\{\left(1,1,1\right),\left(1,1,0\right)\right\}$, $\tau_{\left\{A_{1},A_{3}\right\}}=\left\{\left(1,1,1\right),\left(1,0,1\right)\right\}$, $\tau_{\left\{A_{2},A_{3}\right\}}=\left\{\left(1,1,1\right),\left(0,1,1\right)\right\}$, and $\tau_{\left\{A_{1},A_{2},A_{3}\right\}}=\left(1,1,1\right)$. Hence

$$\begin{array}{c} f\left(\theta ,S\right)=\left(\begin{array}{c} g(\theta_{\tau_{A_{1}}},\left\{A_{1}\right\})\\ g(\theta_{\tau_{A_{2}}},\left\{A_{2}\right\})\\ g(\theta_{\tau_{A_{3}}},\left\{A_{3}\right\})\\ g(\theta_{\tau_{\left\{A_{1},A_{2}\right\}}},\left\{A_{1},A_{2}\right\})\\ g(\theta_{\tau_{\left\{A_{1},A_{3}\right\}}},\left\{A_{1},A_{3}\right\})\\ g(\theta_{\tau_{\left\{A_{2},A_{3}\right\}}},\left\{A_{2},A_{3}\right\})\\ g(\theta_{\tau_{\left\{A_{1},A_{2},A_{3}\right\}}},\left\{A_{1},A_{2},A_{3}\right\}) \end{array}\right)=\left(\begin{array}{c} \theta_{111}+\theta_{110}+\theta_{101}+\theta_{100}\\ \theta_{111}+\theta_{110}+\theta_{011}+\theta_{010}\\ \theta_{111}+\theta_{011}+\theta_{101}+\theta_{001}\\ \theta_{111}+\theta_{110}\\ \theta_{111}+\theta_{101}\\ \theta_{111}+\theta_{011}\\ \theta_{111} \end{array}\right)=\left(\begin{array}{c} \eta_{1}\\ \eta_{2}\\ \eta_{3}\\ \eta_{12}\\ \eta_{13}\\ \eta_{23}\\ \eta_{123} \end{array}\right) \end{array}$$

We are thus computing the frequency of the “marginal” haplotypes over sets of singletons, pairs, and triplets of markers.
Lemma 2
(reparametrization η) Let $\eta ={\left(\eta_{s}\right)}_{s\in S}=f\left(\theta ,S\right)={\left\{g\left(\theta_{\tau_{s}},s\right)\right\}}_{s\in S}$ , with η∈Λ, and Λ={η∣η=f(θ,S),θ∈Θ}. Then, η is a reparametrization of θ. That is, f:Θ→Λ is bijective.

Notice here that we limit η to take values in the image of function *f*. This guarantees that when a bijective function is used to map η back to θ's, those haplotype frequencies will be properly defined, that is $\theta \in {\left(0,1\right)}^{M}$ and $\sum_{h\in H}\theta_{h}$=1. Before we proceed to prove Lemma 2, we introduce the inverse functions of *g* and *f*.

#### Mapping function $g^{-1}$

Let $H^{*}=\left\{v\in {\left\{0,1\right\}}^{M}|\left\langle v,v\right\rangle \neq 0\right\}$ the set of all possible haplotypes over *M* loci except the haplotype containing only “0” alleles. For a haplotype $h\in H^{*}$, let $s_{h}=\left\{s\in S|h\left[j\right]=1\Leftrightarrow A_{j}\in s\right\}$ and $\tau_{h}=\left\{s\in S|s_{h}\subseteq s\right\}$. We define $g^{-1}$ to be a function that takes as an input the parameter vector $\eta ={\left\{\eta_{s}\right\}}_{s\in S}$ and outputs $\theta_{h}$, the frequency of haplotype *h*, for $h\in H^{*}$. That is,

$$\begin{array}{c} g^{-1}\left(\eta ,h\right)=\sum_{s\in \tau_{h}}{\left(-1\right)}^{\left|s_{h}\right|+\left|s\right|}\eta_{s}=\theta_{h}. \end{array}$$

We illustrate $g^{-1}$ with the following example. Let M=3 markers and h=(1,0,0). Then $s_{h}=\left\{A_{1}\right\}$ and $\tau_{h}=\left\{\left\{A_{1},A_{2}\right\},\left\{A_{1},A_{3}\right\},\left\{A_{1},A_{2},A_{3}\right\}\right\}$. Thus $\theta_{100}={\left(-1\right)}^{1}\left\{{\left(-1\right)}^{1}\eta_{1}+{\left(-1\right)}^{2}\eta_{12}+{\left(-1\right)}^{2}\eta_{13}+{\left(-1\right)}^{3}\eta_{123}\right\}=\eta_{1}-\eta_{12}-\eta_{13}+\eta_{123}=\eta_{1}-\left(\eta_{12}-\eta_{123}\right)-\left(\eta_{13}-\eta_{123}\right)-\eta_{123}=\eta_{1}-\theta_{110}-\theta_{101}-\theta_{111}$.

#### Mapping function $f^{-1}$

We define $f^{-1}$ to be a function that takes as input the parameter vector η and outputs the haplotype frequencies θ. That is,

$$\begin{array}{c} f^{-1}\left(\eta ,H\right)=\left[{\left\{g^{-1}\left(\eta_{s_{h}},h\right)\right\}}_{h\in H^{*}},1-\sum_{h\in H^{*}}g^{-1}\left(\eta_{s_{h}},h\right)\right]. \end{array}$$

Notice here that the frequency of the haplotype that contains the “0” allele at all the markers is computed as one minus the sum of the frequencies of all other haplotypes, that is all $h\in H^{*}$. This guarantees that $\sum_{h\in H}\theta_{h}=1$.

We illustrate $f^{-1}$ with the following example. For M=3 markers

$$\begin{array}{c} H^{*}=\left\{(1,0,0),(0,1,0),(0,0,1),(1,1,0),(1,0,1),(0,1,1),(1,1,1)\right\}, \end{array}$$

and

$$\begin{array}{cccc} & s_{\left(1,0,0\right)}=\left\{A_{1}\right\} & \;\text{and}\;\; & \tau_{\left(1,0,0\right)}=\left\{\left\{A_{1}\right\},\left\{A_{1},A_{2}\right\},\left\{A_{1},A_{3}\right\},\left\{A_{1},A_{2},A_{3}\right\}\right\}.\\ & s_{\left(0,1,0\right)}=\left\{A_{2}\right\} & \;\text{and}\;\; & \tau_{\left(0,1,0\right)}=\left\{\left\{A_{2}\right\},\left\{A_{1},A_{2}\right\},\left\{A_{2},A_{3}\right\},\left\{A_{1},A_{2},A_{3}\right\}\right\},\\ & s_{\left(0,0,1\right)}=\left\{A_{3}\right\} & \;\text{and}\;\; & \tau_{\left(0,0,1\right)}=\left\{\left\{A_{3}\right\},\left\{A_{1},A_{3}\right\},\left\{A_{2},A_{3}\right\},\left\{A_{1},A_{2},A_{3}\right\}\right\},\\ & s_{\left(1,1,0\right)}=\left\{A_{1},A_{2}\right\} & \;\text{and}\;\; & \tau_{\left(1,1,0\right)}=\left\{\left\{A_{1},A_{2}\right\},\left\{A_{1},A_{2},A_{3}\right\}\right\},\\ & s_{\left(1,0,1\right)}=\left\{A_{1},A_{3}\right\} & \;\text{and}\;\; & \tau_{\left(1,0,1\right)}=\left\{\left\{A_{1},A_{3}\right\},\left\{A_{1},A_{2},A_{3}\right\}\right\}, \end{array}$$

$$\begin{array}{cccc} & s_{\left(0,1,1\right)}=\left\{A_{2},A_{3}\right\} & \;\text{and}\;\; & \tau_{\left(0,1,1\right)}=\left\{\left\{A_{2},A_{3}\right\},\left\{A_{1},A_{2},A_{3}\right\}\right\},\\ & s_{\left(1,1,1\right)}=\left\{A_{1},A_{2},A_{3}\right\} & \;\text{and}\;\; & \tau_{\left(1,1,1\right)}=\left\{A_{1},A_{2},A_{3}\right\}. \end{array}$$

Hence

$$\begin{array}{c} f^{-1}\left(\eta ,H\right)=\left(\begin{array}{c} \theta_{111}\\ \theta_{011}\\ \theta_{101}\\ \theta_{110}\\ \theta_{001}\\ \theta_{010}\\ \theta_{100}\\ \theta_{000} \end{array}\right)=\left(\begin{array}{c} \eta_{123}\\ \eta_{23}-\eta_{123}\\ \eta_{13}-\eta_{123}\\ \eta_{12}-\eta_{123}\\ \eta_{3}-\eta_{13}-\eta_{23}+\eta_{123}\\ \eta_{2}-\eta_{12}-\eta_{23}+\eta_{123}\\ \eta_{1}-\eta_{12}-\eta_{13}+\eta_{123}\\ 1-\left\{\sum_{h\in H^{*}}g^{-1}\left(\eta_{s_{h}},h\right)\right\} \end{array}\right). \end{array}$$

We now proceed with the proof of Lemma 2. To prove that *f* is bijective we need to show that *f* is both injective, that is $\forall \theta ,\theta^{*}\in \Theta ,f\left(\theta ,H\right)=f\left(\theta^{*},H\right)\Rightarrow \theta =\theta^{*}$, and surjective, that is ∀η∈Λ,∃θ∈Θ:f(θ,H)=η. Now that we have defined the inverse function of *f*, it is easy to show that,

$$\begin{array}{c} f\left(\theta ,H\right)=f\left(\theta^{*},H\right)\Rightarrow f^{-1}\left\{f\left(\theta ,H\right)\right\}=f^{-1}\left\{f\left(\theta^{*},H\right)\right\}\Rightarrow \theta =\theta^{*} \end{array}$$

and ∀ arbitrary parameter vectors η∈Λ we can choose $\theta =f^{-1}\left(\eta ,H\right)$ such that $f\left(\theta ,S\right)=f\{f^{-1}\left(\eta ,H\right),S\}=\eta$. This concludes the proof of the bijectiveness of *f*, which concludes also the proof of Lemma 2.

We now proceed to prove that δ is a reparametrization of η and hence a reparametrization of θ. First, we introduce functions c(η,s) and q(η,S).

#### Mapping function κ

Let $P_{s}$ refer to the family of sets of all possible partitions of a set of random variables s,s∈S, into nonempty subsets (blocks). So, for $p\in P_{s}$, each b∈p is a block. Moreover, let $\tau_{s}^{\prime }=2^{s}\setminus \emptyset$ the power set of *s* minus the empty set. We define κ to be a function that takes as an input the parameter vector $\eta ={\left(\eta_{s}\right)}_{s\in S}$ and outputs the joint cumulant of the set of random variables in *s*, which we denote by $\delta_{s}$. That is,

$$\begin{array}{c} \kappa \left(\eta ,s\right)=\sum_{p\in P_{s}}{\left(-1\right)}^{\left|p\right|-1}\left(\left|p\right|-1\right)!\prod_{b\in p}\text{E}\left(\prod_{A\in b}A\right)=\sum_{p\in P_{s}}{\left(-1\right)}^{\left|p\right|-1}\left(\left|p\right|-1\right)!\prod_{b\in p}\eta_{b}=\delta_{s}. \end{array}$$

We illustrate function *c* with the following example. Let M=3 and $s=\{A_{1},A_{2},A_{3}\}$. Then, $P_{s}=\left\{\left\{A_{1},A_{2},A_{3}\right\},\left\{\left\{A_{1},A_{2}\right\},\left\{A_{3}\right\}\right\},\left\{\left\{A_{1},A_{3}\right\},\left\{A_{2}\right\}\right\},\left\{\left\{A_{2},A_{3}\right\},\left\{A_{1}\right\},\right\},\left\{\left\{A_{1}\right\},\left\{A_{2}\right\},\left\{A_{3}\right\}\right\}\right\}$ and $\eta_{\tau_{s}^{\prime }}=\left(\eta_{1},\eta_{2},\eta_{3},\eta_{12},\eta_{13},\eta_{23},\eta_{123}\right)$. Thus, $\kappa \left(\eta_{\tau_{s}^{\prime }},s\right)={\left(-1\right)}^{1-1}\left(1-1\right)!\;\eta_{\left\{A_{1},A_{2},A_{3}\right\}}+{\left(-1\right)}^{2-1}\left(2-1\right)!\;\eta_{\left\{A_{1},A_{2}\right\}}\eta_{\left\{A_{3}\right\}}+{\left(-1\right)}^{2-1}\left(2-1\right)!\eta_{\left\{A_{1},A_{3}\right\}}\eta_{\left\{A_{2}\right\}}+{\left(-1\right)}^{2-1}\left(2-1\right)!\;\eta_{\left\{A_{2},A_{3}\right\}}\eta_{\left\{A_{1}\right\}}+{\left(-1\right)}^{3-1}\left(3-1\right)!\;\eta_{\left\{A_{1}\right\}}\eta_{\left\{A_{2}\right\}}\eta_{\left\{A_{3}\right\}}=\eta_{123}+\eta_{12}\eta_{3}+\eta_{13}\eta_{2}+\eta_{23}\eta_{1}+2\;\eta_{1}\eta_{2}\eta_{3}$

#### Mapping function q

We define *q* to be a function that takes as input the parameter vector $\eta ={\left(\eta_{s}\right)}_{s\in S}$ and outputs the joint cumulant of random variables in *s* for all s∈S. That is,

$$\begin{array}{c} q\left(\eta ,S\right)={\left\{\kappa \left(\eta_{\tau_{s}^{\prime }},s\right)\right\}}_{s\in S}={\left\{\delta_{s}\right\}}_{s\in S}. \end{array}$$

We illustrate *q* with the following example. For M=3, $P_{\left\{A_{1}\right\}}=\left\{A_{1}\right\}$, $P_{\left\{A_{2}\right\}}=\left\{A_{2}\right\}$, $P_{\left\{A_{3}\right\}}=\left\{A_{3}\right\}$, $P_{\left\{A_{1},A_{2}\right\}}=\{\left\{A_{1},A_{2}\right\},\left\{\left\{A_{1}\right\},\left\{A_{2}\right\}\right\}$, $P_{\left\{A_{1},A_{3}\right\}}=\{\left\{A_{1},A_{3}\right\},\left\{\left\{A_{1}\right\},\left\{A_{3}\right\}\right\}$, $P_{\left\{A_{2},A_{3}\right\}}=\{\left\{A_{2},A_{3}\right\},\left\{\left\{A_{2}\right\},\left\{A_{3}\right\}\right\}$, and $P_{\left\{A_{1},A_{2},A_{3}\right\}}=\left\{\left\{A_{1},A_{2},A_{3}\right\},\left\{\left\{A_{1},A_{2}\right\},\left\{A_{3}\right\}\right\},\left\{\left\{A_{1},A_{3}\right\},\left\{A_{2}\right\}\right\},\left\{\left\{A_{2},A_{3}\right\},\left\{A_{1}\right\}\right\},\left\{\left\{A_{1}\right\},\left\{A_{2}\right\},\left\{A_{3}\right\}\right\}\right\}$.

Hence $q\left(\eta ,S\right)=\left(\begin{array}{c} \eta_{1}\\ \eta_{2}\\ \eta_{3}\\ \eta_{12}-\eta_{1}\eta_{2}\\ \eta_{13}-\eta_{1}\eta_{3}\\ \eta_{23}-\eta_{2}\eta_{3}\\ \eta_{123}-\eta_{12}\eta_{3}-\eta_{13}\eta_{2}-\eta_{23}\eta_{1}+2\eta_{1}\eta_{2}\eta_{3} \end{array}\right)$.

We are thus computing the joint cumulant of all possible sets of singletons, pairs, and triplets of markers.
Lemma 3
(reparametrization δ) Let $\delta ={\left(\delta_{s}\right)}_{s\in S}=q\left(\eta ,S\right)={\left\{\kappa \left(\eta_{\tau_{s}^{\prime }},s\right)\right\}}_{s\in S}$, with δ∈Δ and Δ={δ∣δ=q∘f(θ,S),θ∈Θ}. Then δ is a reparametrization of η. That is, q:Λ→Δ is a bijective mapping function.

Notice here that we limit δ to take values in the image of function *q*. This guarantees that when a bijective function is used to map δ back to η and then back to θ's, those haplotype frequencies will be properly defined. Before we proceed to prove Lemma 3, we introduce the inverse functions of *c* and *q*.

#### Mapping function $\kappa^{-1}$

We define $\kappa^{-1}$ to be a function that takes as an input the parameter vector $\delta ={\left(\delta_{s}\right)}_{s\in S}$ and outputs $\eta_{s}$, the joint expectation of the set of random variables in *s*. That is,

$$\begin{array}{c} \kappa^{-1}\left(\delta ,s\right)=\delta_{s}-\sum_{p\in P_{s}\setminus s}{\left(-1\right)}^{\left|p\right|}\left(\left|p\right|-1\right)!\prod_{b\in p}\left\{\delta_{b}-\sum_{p^{\prime }\in P_{b}\setminus b}{\left(-1\right)}^{\left|p^{\prime }\right|}\left(\left|p^{\prime }\right|-1\right)!\prod_{b^{\prime }\in p^{\prime }}\delta_{b^{\prime }}\right\}. \end{array}$$

We illustrate function $\kappa^{-1}$ with the following example. Let M=3 and $s=\{A_{1},A_{2},A_{3}\}$. Then, $\delta_{\tau_{s}^{\prime }}=\left(\delta_{1},\delta_{2},\delta_{3},\delta_{12},\delta_{13},\delta_{23},\delta_{123}\right)$. Hence,

$$\begin{array}{ccc} \kappa^{-1}\left(\delta_{\tau_{s}^{\prime }},s\right) & = & \delta_{\left\{A_{1},A_{2},A_{3}\right\}}+{\left(-1\right)}^{2}\left(2-1\right)!\;\left(\delta_{\left\{A_{1},A_{2}\right\}}+{\left(-1\right)}^{2}\left(2-1\right)!\;\delta_{A_{1}}\delta_{A_{2}}\right)\delta_{A_{3}}\\ & & +\;{\left(-1\right)}^{2}\left(2-1\right)!\;\left(\delta_{\left\{A_{1},A_{3}\right\}}+{\left(-1\right)}^{2}\left(2-1\right)!\;\delta_{A_{1}}\delta_{A_{3}}\right)\delta_{A_{2}}\\ & & +\;{\left(-1\right)}^{2}\left(2-1\right)!\;\left(\delta_{\left\{A_{2},A_{3}\right\}}+{\left(-1\right)}^{2}\left(2-1\right)!\;\delta_{A_{2}}\delta_{A_{3}}\right)\delta_{A_{1}}\\ & & +\;{\left(-1\right)}^{3}\left(3-1\right)!\;\delta_{A_{1}}\delta_{A_{2}}\delta_{A_{3}}\\ & = & \delta_{\left\{A_{1},A_{2},A_{3}\right\}}+\left(\delta_{\left\{A_{1},A_{2}\right\}}+\delta_{A_{1}}\delta_{A_{2}}\right)\delta_{A_{3}}+\left(\delta_{\left\{A_{1},A_{3}\right\}}+\delta_{A_{1}}\delta_{A_{3}}\right)\delta_{A_{2}}\\ & & +\;\left(\delta_{\left\{A_{2},A_{3}\right\}}+\delta_{A_{2}}\delta_{A_{3}}\right)\delta_{A_{1}}-2\delta_{A_{1}}\delta_{A_{2}}\delta_{A_{3}} \end{array}$$

Which is the same expression we would get if we used the definition of $\delta_{\left\{A_{1},A_{2},A_{3}\right\}}$, and solved for η_123_, that is $\eta_{123}=\delta_{\left\{A_{1},A_{2},A_{3}\right\}}+\eta_{12}\eta_{3}+\eta_{13}\eta_{2}+\eta_{23}\eta_{1}-2\eta_{1}\eta_{2}\eta_{3}=\delta_{\left\{A_{1},A_{2},A_{3}\right\}}+\left(\delta_{\left\{A_{1},A_{2}\right\}}+\delta_{A_{1}}\delta_{A_{2}}\right)\delta_{A_{3}}+\left(\delta_{\left\{A_{1},A_{3}\right\}}+\delta_{A_{1}}\delta_{A_{3}}\right)\delta_{A_{2}}+\left(\delta_{\left\{A_{2},A_{3}\right\}}+\delta_{A_{2}}\delta_{A_{3}}\right)\delta_{A_{1}}-2\delta_{A_{1}}\delta_{A_{2}}\delta_{A_{3}}$.

For a set $\left\{A_{1},A_{2},A_{3}\right\}$ of random variables, we will write δ_123_ as a shorthand of $\delta_{\left\{A_{1},A_{2},A_{3}\right\}}$.

#### Mapping function $q^{-1}$

We define $q^{-1}$ to be a function that takes as input the parameter vector $\delta ={\left(\delta_{s}\right)}_{s\in S}$ and outputs $\eta ={\left(\eta_{s}\right)}_{s\in S}$, the joint expectation of the set of random variables in *s* for all s∈S. That is,

$$\begin{array}{c} q^{-1}\left(\delta ,S\right)={\left\{\kappa^{-1}\left(\delta_{\tau_{s}^{\prime }},s\right)\right\}}_{s\in S}={\left(\eta_{s}\right)}_{s\in S}. \end{array}$$

We illustrate $q^{-1}$ with the following example. For M=3,

$$\begin{array}{c} q^{-1}\left(\delta ,S\right)=\left(\begin{array}{c} \delta_{1}\\ \delta_{2}\\ \delta_{3}\\ \delta_{12}+\delta_{1}\delta_{2}\\ \delta_{13}+\delta_{1}\delta_{3}\\ \delta_{23}+\delta_{2}\delta_{3}\\ \delta_{123}+\delta_{12}\delta_{3}+\delta_{13}\delta_{2}+\delta_{23}\delta_{1}-2\delta_{1}\delta_{2}\delta_{3} \end{array}\right)=\left(\begin{array}{c} \eta_{1}\\ \eta_{2}\\ \eta_{3}\\ \eta_{12}\\ \eta_{13}\\ \eta_{23}\\ \eta_{123} \end{array}\right). \end{array}$$

We now proceed with the proof of Lemma 3. To prove that *q* is bijective we need to show that *q* is both injective, that is $\forall \eta ,\eta^{*}\in \Lambda ,q\left(\eta ,S\right)=q\left(\eta^{*},S\right)\Rightarrow \eta =\eta^{*}$, and surjective, that is ∀δ∈Δ,∃η∈Λ:q(η,S)=δ. Now that we have defined the inverse function of *q*, it is easy to show that, $q\left(\eta ,S\right)=q\left(\eta^{*},S\right)\Rightarrow q^{-1}\left\{q\left(\eta ,S\right)\right\}=q^{-1}\left\{q\left(\eta^{*},S\right)\right\}\Rightarrow \eta =\eta^{*}$ and for all arbitrary parameter vectors δ∈Δ, we can choose $\eta =q^{-1}\left(\delta ,S\right)$ such that $q\left(\eta ,S\right)=q\{q^{-1}\left(\delta ,S\right),S\}=\delta$. This concludes the proof of the bijectiveness of *q*, which concludes also the proof of Lemma 3 and thus of Lemma 1.

### A.2 Standardized parameters

Recall that $\delta_{A}$ can be expressed as

$$\begin{array}{c} \delta_{A}=\eta_{A}-\sum_{p\in P_{A}\setminus A}{\left(-1\right)}^{\left|p\right|}\left(\left|p\right|-1\right)!\prod_{b\in p}\eta_{b}=\eta_{A}-\sum_{p\in P_{A}\setminus A}R_{\delta }\left(p\right)=\eta_{A}-R_{\delta } \end{array}$$

where $R_{\delta }\left(p\right)$'s depend on loci b∈p with |b|<M. These rest terms $R_{\delta }\left(p\right)$ are considered fixed and bounds for $\delta_{A}$ are to be determined completely analogous to the two locus case based on $\eta_{A}$.

First, $\eta_{A}$ is upper bound by all lower order $\eta_{s}$ and lower bound by 0. That is

$$\begin{array}{cc} \eta_{A} & \leq U_{1}\left(A\right):=\min\left\{\eta_{s}|s\in S\setminus A\right\}.\\ \eta_{A} & \geq L_{1}\left(A\right)=0 \end{array}$$

Second, further constraints are imposed by the relationship between $\eta_{A}$ and lower order haplotype frequencies $\eta_{s}$. It is straightforward to see that $\eta_{s}$ can be restated as:

$$\begin{array}{cc} & \;\eta_{s}=g\left(\theta ,s\right)=\theta_{h_{s}}+\sum_{t\in S,t⊃s}{\left(-1\right)}^{\left|t\right|-\left|s\right|-1}\eta_{t} \end{array}$$

Here, $\theta_{h_{s}}$ is the frequency for haplotype $h_{s}=\left\{v\in {\left\{0,1\right\}}^{M}|v\left[j\right]\Leftrightarrow A_{j}\in s\right\}$, the haplotype with *M* loci with 1‐alleles at loci *s* and 0‐alleles elsewhere. Note that all the sums above include $\eta_{A}$. Solving for $\eta_{A}$ gives us:

$$\begin{array}{cc} \eta_{A} & ={\left(-1\right)}^{\left|A\right|-\left|s\right|-1}\left\{\eta_{s}-\theta_{h_{s}}-\sum_{t\in S\setminus A,t⊃s}{\left(-1\right)}^{\left|t\right|-\left|s\right|-1}\eta_{t}\right\}\\ & ={\left(-1\right)}^{\left|A\right|-\left|s\right|}\left\{\theta_{h_{s}}-\sum_{t\in S\setminus A,t⊇s}{\left(-1\right)}^{\left|t\right|-\left|s\right|}\eta_{t}\right\}\\ & =\sigma_{s}\left(\theta_{h_{s}}-R_{s}\right), \end{array}$$

where $\sigma_{s}={\left(-1\right)}^{\left|A|-|s\right|}$ and $R_{s}=\sum_{t\in S\setminus A,t⊇s}{\left(-1\right)}^{\left|t|-|s\right|}\eta_{t}$. Each $\eta_{s}$ therefore contributes an upper and lower bound to $\eta_{A}$ by choosing $\theta_{h_{s}}=0$ or $\theta_{h_{s}}=1$:

$$\begin{array}{cc} \eta_{A}\leq U_{s} & :=\left\{\begin{array}{cc} \max(1-R_{s},0) & \text{if}\;\sigma_{s}\geq 0\;\text{and}\;\\ \min(-R_{s},0) & \text{if}\;\sigma_{s}<0, \end{array}\right. \end{array}$$

and

$$\begin{array}{cc} \eta_{A}\geq L_{s} & :=\left\{\begin{array}{cc} \max(-R_{s},0) & \text{if}\;\sigma_{s}\geq 0\;\text{and}\;\\ \min(1-R_{s},0) & \text{if}\;\sigma_{s}<0 \end{array}\right.. \end{array}$$

With $U_{2}\left(A\right):=\min\left\{U_{s}|s\in S\setminus A\right\}$ and $L_{2}\left(A\right):=\max\left\{L_{s}|s\in S\setminus A\right\}$, we get

$$\begin{array}{c} \eta_{A}^{\max}:=\min\left\{U_{1}\left(A\right),U_{2}\left(A\right)\right\},\\ \eta_{A}^{\min}:=\max\left\{L_{1}\left(A\right),L_{2}\left(A\right)\right\}. \end{array}$$

Then $\eta_{A}^{\max}$ and $\eta_{A}^{\min}$ can be used as above to standardize $\delta_{A}$.

### A.3 Additional Tables

**Table A.1:** Linkage disequilibrium parameters in the cases (Ca), controls (Co), and pool (P) of cases and controls samples for each of the four triplets identified from the WTCCC data analysis

	Triplet 1			Triplet 2			Triplet 3			Triplet 4		
	P	Ca	Co	P	Ca	Co	P	Ca	Co	P	Ca	Co
δ_1_	0.239	0.264	0.223	0.239	0.264	0.223	0.350	0.324	0.366	0.213	0.234	0.199
δ_2_	0.126	0.120	0.130	0.091	0.108	0.081	0.207	0.191	0.218	0.401	0.376	0.417
δ_3_	0.091	0.108	0.081	0.314	0.308	0.319	0.247	0.276	0.229	0.199	0.209	0.193
δ_12_	−0.374	−0.502	−0.279	0.111	0.135	0.081	0.712	0.696	0.720	−0.297	−0.268	−0.306
δ_13_	0.111	0.135	0.081	−0.112	−0.199	−0.046	0.103	0.033	0.170	−0.759	−0.790	−0.739
δ_23_	−0.544	−0.382	−0.663	0.363	0.351	0.377	−0.105	−0.174	−0.040	0.227	0.088	0.335
δ_123_	0.172	0.084	0.229	−0.697	−0.665	−0.716	−0.160	−0.119	−0.190	0.203	0.521	−0.126

**Table A.2:** Corresponding haplotype frequencies for SNPs in linkage equilibrium (Scenario 1), and SNPs in LD (Scenario 2)

Haplotype	Scenario 1	Scenario 2
θ_0000_	0.292	0.416
θ_0001_	0.239	0.183
θ_0010_	0.135	0.066
θ_0011_	0.111	0.126
θ_0100_	0.065	0.022
θ_0101_	0.054	0.042
θ_0110_	0.030	0.029
θ_0111_	0.025	0.065
θ_1000_	0.015	0.001
θ_1001_	0.013	0.008
θ_1010_	0.007	0.006
θ_1011_	0.006	0.010
θ_1100_	0.003	0.007
θ_1101_	0.003	0.005
θ_1110_	0.002	0.003
θ_1111_	0.001	0.011
