# Supplementary material for: Powerful testing via hierarchical linkage disequilibrium in haplotype association studies
Source: Biom J. 2019 Jan 28;61(3):747–68. doi: 10.1002/bimj.201800053 (PMC6637384; doi:10.1002/bimj.201800053)
Supplement: Supplementary file 1 — Supporting Information [file BIMJ-61-747-s001.zip › reproducibility/README.pdf]

Title: Powerful testing via hierarchical linkage disequilibrium in haplotype association studies

Authors: Brunilda Balliu, Jeanine J Houwing-Duistermaat, and Stefan Boehringer

Code provided by: Brunilda Balliu (bballiu@stanford.edu) and Stefan Boehringer (correspondence@s-boehringer.org)

Date: November 2018

The code has been tested under the following environment (case_study2).

```
R version 3.5.0 (2018-04-23)
Platform: x86_64-pc-linux-gnu (64-bit)
Running under: openSUSE Leap 42.3

Matrix products: default
BLAS: /home/pingu/lib64/R-3.5.0/lib/libRblas.so
LAPACK: /home/pingu/lib64/R-3.5.0/lib/libRlapack.so

locale:
 [1] LC_CTYPE=en_US.utf-8       LC_NUMERIC=C
 [3] LC_TIME=en_US.utf-8        LC_COLLATE=en_US.utf-8
 [5] LC_MONETARY=en_US.utf-8    LC_MESSAGES=en_US.utf-8
 [7] LC_PAPER=en_US.utf-8       LC_NAME=C
 [9] LC_ADDRESS=C               LC_TELEPHONE=C
[11] LC_MEASUREMENT=en_US.utf-8 LC_IDENTIFICATION=C

attached base packages:
[1] parallel  tools     stats     graphics  grDevices datasets  utils
[8] methods   base

other attached packages:
 [1] WriteXLS_4.0.0         wCorr_1.9.1            ggplot2_2.2.1
 [4] gtools_3.8.1           numDeriv_2016.8-1      partitions_1.9-19
 [7] sets_1.0-18            haplo.stats_1.7.9      snpStats_1.30.0
[10] Matrix_1.2-14          survival_2.41-3        parallelize.dynamic_1.0

loaded via a namespace (and not attached):
 [1] zoo_1.8-2          splines_3.5.0      lattice_0.20-35
 [4] colorspace_1.3-2   htmltools_0.3.6    base64enc_0.1-4
```

```
 [7] gmp_0.5-13.2        rlang_0.3.0.1       pillar_1.2.3
[10] foreign_0.8-70      BiocGenerics_0.26.0 RColorBrewer_1.1-2
[13] multcomp_1.4-8      plyr_1.8.4          stringr_1.3.1
[16] zlibbioc_1.26.0     MatrixModels_0.4-1  munsell_0.5.0
[19] gtable_0.2.0        htmlwidgets_1.2     mvtnorm_1.0-8
[22] codetools_0.2-15    labeling_0.3        latticeExtra_0.6-28
[25] knitr_1.20          SparseM_1.77        quantreg_5.36
[28] htmlTable_1.12      TH.data_1.0-8       Rcpp_0.12.17
[31] acepack_1.4.1       polynom_1.3-9       backports_1.1.2
[34] scales_0.5.0        checkmate_1.8.5     rms_5.1-2
[37] Hmisc_4.1-1         gridExtra_2.3       mnormt_1.5-5
[40] digest_0.6.15       stringi_1.2.3       polspline_1.1.13
[43] grid_3.5.0          sandwich_2.4-0      magrittr_1.5
[46] lazyeval_0.2.1      tibble_1.4.2        Formula_1.2-3
[49] cluster_2.0.7-1     MASS_7.3-49         data.table_1.11.4
[52] minqa_1.2.4         rstudioapi_0.7      rpart_4.1-13
[55] nnet_7.3-12         nlme_3.1-137        compiler_3.5.0
```

For `simulations` and `case_study1`, the following environment was used.

```
R version 3.5.0 (2018-04-23)
Platform: x86_64-pc-linux-gnu (64-bit)
Running under: openSUSE Leap 42.3

Matrix products: default
BLAS: /home/pingu/lib64/R-3.5.0/lib/libRblas.so
LAPACK: /home/pingu/lib64/R-3.5.0/lib/libRlapack.so

locale:
 [1] LC_CTYPE=en_US.utf-8       LC_NUMERIC=C
 [3] LC_TIME=en_US.utf-8        LC_COLLATE=en_US.utf-8
 [5] LC_MONETARY=en_US.utf-8    LC_MESSAGES=en_US.utf-8
 [7] LC_PAPER=en_US.utf-8       LC_NAME=C
 [9] LC_ADDRESS=C               LC_TELEPHONE=C
[11] LC_MEASUREMENT=en_US.utf-8 LC_IDENTIFICATION=C

attached base packages:
[1] stats     graphics  grDevices datasets  utils     methods   base

other attached packages:
 [1] PropCIs_0.3-0       boot_1.3-20         haplo.stats_1.7.9
 [4] HardyWeinberg_1.6.1 Rsolnp_1.16         mice_3.3.0
 [7] lattice_0.20-35     snpStats_1.30.0     Matrix_1.2-14
[10] survival_2.41-3     numDeriv_2016.8-1   partitions_1.9-19
[13] sets_1.0-18

loaded via a namespace (and not attached):
```

```
 [1] tidyr_0.8.2         splines_3.5.0       Formula_1.2-3
 [4] assertthat_0.2.0    latticeExtra_0.6-28 pillar_1.2.3
 [7] backports_1.1.2     quantreg_5.36       glue_1.3.0
[10] digest_0.6.15       RColorBrewer_1.1-2  checkmate_1.8.5
[13] minqa_1.2.4         colorspace_1.3-2    sandwich_2.4-0
[16] rms_5.1-2           htmltools_0.3.6     plyr_1.8.4
[19] pkgconfig_2.0.1     broom_0.5.1         SparseM_1.77
[22] zlibbioc_1.26.0     purrr_0.2.5         mvtnorm_1.0-8
[25] scales_0.5.0        lme4_1.1-17         MatrixModels_0.4-1
[28] htmlTable_1.12      tibble_1.4.2        gmp_0.5-13.2
[31] generics_0.0.2      ggplot2_2.2.1       TH.data_1.0-8
[34] pan_1.6             nnet_7.3-12         BiocGenerics_0.26.0
[37] lazyeval_0.2.1      magrittr_1.5        mitml_0.3-6
[40] polspline_1.1.13    nlme_3.1-137        MASS_7.3-49
[43] foreign_0.8-70      truncnorm_1.0-8     tools_3.5.0
[46] data.table_1.11.4   multcomp_1.4-8      stringr_1.3.1
[49] munsell_0.5.0       cluster_2.0.7-1     bindrcpp_0.2.2
[52] compiler_3.5.0      rlang_0.3.0.1       grid_3.5.0
[55] nloptr_1.0.4        rstudioapi_0.7      htmlwidgets_1.2
[58] base64enc_0.1-4     gtable_0.2.0        codetools_0.2-15
[61] polynom_1.3-9       R6_2.2.2            gridExtra_2.3
[64] zoo_1.8-2           knitr_1.20          dplyr_0.7.5
[67] bindr_0.1.1         Hmisc_4.1-1         jomo_2.6-5
[70] stringi_1.2.3       parallel_3.5.0      Rcpp_0.12.17
[73] rpart_4.1-13        acepack_1.4.1       tidyselect_0.2.5
```

Folder structure

- `simulation`: directory with simulation study (intermediate) results
  - `intermediate_results`: contains pre-computed intermediate results
    - `j.txt` : simulation results for the first *1,000* iterations of scenario j. Parameter values for j can be found in `Run_simulation_study.R`
    - `Table*_manuscript.txt`: Tables 2 - 5 from manuscript. Based on *10,000* iterations
  - `code`: directory with shared source code for simulation study and case_study1
    - `HierarchicalLD.R`: Functions for all re-parametrizations, likelihoods, and optimization.
    - `Functions_for_simulation_study.R` : Functions for simulating data and computing type I error and power rates
  - `Run_simulation_study.R`: Code for running simulation study and generating Tables 2-5 in the manuscript
- `case_study1_cadidate_loci_analysis`: directory with data and results for candidate loci analysis.

  - `data`: This folder contains instructions on how to obtain the data. Due to privacy restrictions data cannot be distributed with this source package
  - `results`: results from candidate loci analysis, used to produce Table 6
    * `j.Pvalues.txt` contain p-values for Full, P={1}, P={1,2}, and three single SNP tests for each SNP `j` that had at least one triplet with <.8 LD
    * `j.MostSignificantPvalues.txt` contain p-values for all triplets that passed genome-wide significance. Table 6 lists results for j=5, 13, and 20.
  - `code`: shared code with ‘simulation, see above
  - `Run_candidate_loci_analysis.R`: Functions for running candidate_loci__analysis and summarizing results
- `case_study2_genome_wide_analysis`: directory with data and results for the GWAS
  - `data`: This folder contains instructions on how to obtain the data. Due to privacy restrictions data cannot be distributed with this source package
  - `intermediate_results`: pre-results from from the GWAS, used to produce Tables 7, 8, figures 1, 2
  - `src`: R-files with helper functions, the code for the study at hand is contained in the file `HLD.R`
  - `case_study2.R`: Functions for running the GWAS and summarizing results

Simulation study (Tables 2-5)

To run the simulations set your working directory to the folder `simulation` and source the file `Run_simulation_study.R`. Missing packages are reported and have to be installed manually. In the simulation study, we considered 22 different scenarios. For each scenario, we simulated 10,000 data sets (iterations), estimated parameter values, and computed p-values for the differences in haplotype frequencies between cases and controls. We run all simulations using a server.

Since optimizing the likelihood is computationally intensive, we have included results for a sub-sample of *1,000* iterations of each simulation scenario. Note that the tables generated from these files will differ slightly from the tables in the manuscript which are based on *10,000* iterations. Moreover, the results for the *MinPvalKim* will be very different from the ones in the manuscript as they are based on Bonferroni adjusted p-values; the *MinPvalKim* results from the manuscript are based on nominal p-values (as described in the methods).

1. Open R on the project directory.

2. Download and install all the R packages in the beginning of the files `code/HierarchicalLD.R` and `code/Functions_for_simulation_study.R`
3. To run the simulation study and not make use of our intermediate results,

open `code/Run_simulation_study.R` and set `UseIntermediateResults = F` in line 12.

- If running in a single machine (not advised), run the remaining of the code.
- If running on a server, copy the code inside `if(!UseIntermediateResults){ ... }` lines 30-151 and parallelize according to your server. The easiest way is to parallelize each scenario and every couple 100 iterations. Once finished, concatenated all iterations from each scenario in a separate file called `j.txt`, where j is scenario number.
- In the end you should have 22 txt files, one for each scenario, with 10,000 lines each, one line for each iteration. The number of columns in each txt file will differ according to the number of markers.
- Once you have all these results, copy them to the `simulation_study/results/` folder. Note that this will over-write our intermediate results. Then run the remaining of the code in `code/Run_simulation_study.R` to generate the tables.

4. If you do not want to run the simulation study, you can make use of our intermediate results by setting `UseIntermediateResults = T`.

`Table2-5` will contain results generated from your simulation study (`UseIntermediateResults = F`) or using our intermediate results

(`UseIntermediateResults = T`). `Table2-5_manuscript` contains the results from the tables in the manuscript.

Candidate loci analysis (Table 6)

To run the simulations set your working directory to the folder `case_study1_cadidate_loci_analysis` and source the file `RRun_candidate_loci_analysis.R`. Missing packages are reported and have to be installed manually.

To fully re-run the candidate loci analysis, you need to download and process WTCCC RA data as described in the manuscript and under the `data` subfolder.

You should have the following Plink binary file data when you are done: `data/SNP_NAME.Cases.bed/bim/fam, SNP_NAME.Controls.bed/bim/fam` where SNP_NAME indicates the name of each of the 54 candidate loci.

Each candidate loci file will contain genotype data for the candidate loci and it's 40 neighbors, 20 on each side, with the candidate loci in position 21.

Since we cannot provide data, we include intermediate results from the optimization.

To make use of our intermediate results, open R in the project directory and run `code/Run_data_analysis.R` setting `UseIntermediateResults = T` in line 12, otherwise download and process the data and set `UseIntermediateResults = F`.

Genome-wide analysis (Tables 7-8, Figures 1-2)

The genome wide analysis can be run from the `case_study2_genome_wide_analysis` subfolder by setting the working directory to this directory and sourcing the file `case_study2.R`. Required packages are install automatically and require a working internet connection. The analysis is controlled by the following variables.

- `AnalyzeRealData`: boolean to indicate whether real or simulated data is used
- `UseIntermediateResults`: boolean to control in the case of simulations whether a pre-computed simulation should be used. The same results are computing by setting this variable to `FALSE` as a fixed seed is chosen.
- `CreateOutput`: boolean to control whether output files should be saved. By default they are saved into the temporary R folder
- `outputDir`: alternative destination for the output files
- `DoParallelize`: whether to parallelize computations. If set to `TRUE` this requires further configuration to describe the compute resources. This is further explained in the source file.

Due to privacy restrictions the data used for this case study cannot be distributed with this source package. Data is available from the EGA archive. Details of how to acquire the data and how to convert raw files into files usable for analysis are explained in detail in the `data` subfolder. The analyes on simulated data reproduce the structure of the tables and figures exactly but use a simplified simulation without genetic associations. They are meant to illustrate and document the steps taken in the analysis. The caption of the tables has been changed slightly to accomodate the simulations as compared to the paper. Apart from this difference, the code is unchanged.
