# Supplementary material for: Powerful testing via hierarchical linkage disequilibrium in haplotype association studies
Source: Biom J. 2019 Jan 28;61(3):747–68. doi: 10.1002/bimj.201800053 (PMC6637384; doi:10.1002/bimj.201800053)
Supplement: Supplementary file 1 — Supporting Information [file BIMJ-61-747-s001.zip › reproducibility/case_study2_genome_wide_analysis/data/README.pdf]

Data case study2 (GWAS), manuscript “Powerful testing via hierarchical linkage disequilibrium in haplotype association studies”

Data download

Due to privacy restrictions the data used for this case study cannot be distributed with this source package. It can be acquired through the EGA archive (https://ega-archive.org) under the identifier: EGAD00010000929

The data set can be downloaded as follows (assuming a UNIX environment).

Start the EGA client, assuming it is located in the folder /opt/ega.

```
java -jar /opt/ega/EgaDemoClient.jar
```

In the ega client environment execute the following statement to download and decrypt the data.

```
login
request dataset EGAD00010000929 egaDataDownloadSB request_1
download request_1

 decrypt cases.4.bnt.cip egaDataDownloadSB
 decrypt cases.1.bnt.cip egaDataDownloadSB
 decrypt cases.16.bed.cip egaDataDownloadSB
 decrypt cases.1.bed.cip egaDataDownloadSB
 decrypt cases.6.bim.cip egaDataDownloadSB
 decrypt cases.9.bed.cip egaDataDownloadSB
 decrypt cases.21.bnt.cip egaDataDownloadSB
 decrypt cases.10.bim.cip egaDataDownloadSB
 decrypt cases.13.bnt.cip egaDataDownloadSB
 decrypt cases.19.bed.cip egaDataDownloadSB
 decrypt cases.19.bnt.cip egaDataDownloadSB
 decrypt cases.7.bnt.cip egaDataDownloadSB
 decrypt cases.13.bim.cip egaDataDownloadSB
 decrypt cases.11.bim.cip egaDataDownloadSB
 decrypt cases.14.bim.cip egaDataDownloadSB
 decrypt cases.7.bim.cip egaDataDownloadSB
 decrypt cases.3.bim.cip egaDataDownloadSB
 decrypt cases.3.bed.cip egaDataDownloadSB
 decrypt cases.1.bim.cip egaDataDownloadSB
 decrypt cases.17.bim.cip egaDataDownloadSB
 decrypt cases.11.bed.cip egaDataDownloadSB
 decrypt cases.8.bim.cip egaDataDownloadSB
 decrypt cases.14.bed.cip egaDataDownloadSB
```

```
decrypt cases.15.bed.cip egaDataDownloadSB
decrypt cases.20.bed.cip egaDataDownloadSB
decrypt cases.22.bnt.cip egaDataDownloadSB
decrypt cases.16.bnt.cip egaDataDownloadSB
decrypt cases.7.bed.cip egaDataDownloadSB
decrypt cases.5.bed.cip egaDataDownloadSB
decrypt cases.9.bnt.cip egaDataDownloadSB
decrypt cases.4.bed.cip egaDataDownloadSB
decrypt cases.21.bim.cip egaDataDownloadSB
decrypt cases.18.bnt.cip egaDataDownloadSB
decrypt cases.16.bim.cip egaDataDownloadSB
decrypt cases.10.bed.cip egaDataDownloadSB
decrypt cases.8.bed.cip egaDataDownloadSB
decrypt cases.14.bnt.cip egaDataDownloadSB
decrypt cases.21.bed.cip egaDataDownloadSB
decrypt cases.9.bim.cip egaDataDownloadSB
decrypt cases.15.bnt.cip egaDataDownloadSB
decrypt cases.12.bnt.cip egaDataDownloadSB
decrypt cases.19.bim.cip egaDataDownloadSB
decrypt cases.10.bnt.cip egaDataDownloadSB
decrypt cases.2.bnt.cip egaDataDownloadSB
decrypt cases.20.bnt.cip egaDataDownloadSB
decrypt cases.12.bed.cip egaDataDownloadSB
decrypt cases.12.bim.cip egaDataDownloadSB
decrypt cases.5.bnt.cip egaDataDownloadSB
decrypt cases.22.bed.cip egaDataDownloadSB
decrypt cases.4.bim.cip egaDataDownloadSB
decrypt cases.6.bnt.cip egaDataDownloadSB
decrypt cases.11.bnt.cip egaDataDownloadSB
decrypt cases.17.bed.cip egaDataDownloadSB
decrypt cases.6.bed.cip egaDataDownloadSB
decrypt cases.22.bim.cip egaDataDownloadSB
decrypt cases.18.bed.cip egaDataDownloadSB
decrypt cases.2.bim.cip egaDataDownloadSB
decrypt cases.8.bnt.cip egaDataDownloadSB
decrypt cases.fam.cip egaDataDownloadSB
decrypt cases.2.bed.cip egaDataDownloadSB
decrypt cases.15.bim.cip egaDataDownloadSB
decrypt cases.20.bim.cip egaDataDownloadSB
decrypt cases.5.bim.cip egaDataDownloadSB
decrypt cases.3.bnt.cip egaDataDownloadSB
decrypt cases.17.bnt.cip egaDataDownloadSB
decrypt cases.18.bim.cip egaDataDownloadSB
decrypt cases.13.bed.cip egaDataDownloadSB
```

The above data is for cases which were compared to controls of the WTCCC2

study. These can be downloaded in similar fashion under the EGA id EGAD00000000022.

Data merging

Merging case files

Data for cases is split into many files. They can be merged into a single plink file as follows:

```
# create fam files
ls | grep bed | perl -ne '/(.*).bed/; print "$1\n";' | \
  perl -e '@files = map { substr($_, 0, -1) } (<>);
  for $f (@files) { system("ln -s _cases.fam $f.fam") }'
# manual change in _cases.19.bim
#19 rs34536443  0   10324118    G   C
#-->19  rs34536443_1    0   10324118    G   C
# merge
ls | grep bed | perl -ne '/(.*).bed/; print "$1\n";' | perl -e \
  '@files = map { substr($_, 0, -1) } (<>); $inter = shift @files; \
  for $f (@files) { $cmd = "plink --noweb --bfile $inter
  --bmerge $f.bed $f.bim $f.fam --make-bed
  --out interm_$f"; print("$cmd\n"); system($cmd); $inter = "interm_$f"; }
  $cmd = "plink --noweb --bfile $inter --make-bed --out merged"; print("$cmd\n");
  system($cmd)'

plink --bfile data1 --merge data2.ped data2.map --make-bed --out merge

plink --noweb --bfile '_cases.10' --bmerge _cases.11.bed _cases.11.bim _cases.11.fam \
  --make-bed --out 'conversion/_cases'
plink --noweb --bfile 'conversion/_cases' \
  --bmerge _cases.12.bed _cases.12.bim _cases.12.fam --make-bed --out 'conversion/_cases'
plink --noweb --bfile 'conversion/_cases' \
  --bmerge_cases.13.bed _cases.13.bim _cases.13.fam --make-bed --out 'conversion/_cases'
plink --noweb --bfile 'conversion/_cases' \
  --bmerge_cases.14.bed _cases.14.bim _cases.14.fam --make-bed --out 'conversion/_cases'
plink --noweb --bfile 'conversion/_cases' \
  --bmerge_cases.15.bed _cases.15.bim _cases.15.fam --make-bed --out 'conversion/_cases'
plink --noweb --bfile 'conversion/_cases' \
  --bmerge_cases.16.bed _cases.16.bim _cases.16.fam --make-bed --out 'conversion/_cases'
plink --noweb --bfile 'conversion/_cases' \
  --bmerge_cases.17.bed _cases.17.bim _cases.17.fam --make-bed --out 'conversion/_cases'
plink --noweb --bfile 'conversion/_cases' \
  --bmerge_cases.18.bed _cases.18.bim _cases.18.fam --make-bed --out 'conversion/_cases'
plink --noweb --bfile 'conversion/_cases' \
```

```
  --bmerge_cases.19.bed _cases.19.bim _cases.19.fam --make-bed --out 'conversion/_cases'
plink --noweb --bfile 'conversion/_cases' \
  --bmerge_cases.1.bed _cases.1.bim _cases.1.fam --make-bed --out 'conversion/_cases'
plink --noweb --bfile 'conversion/_cases' \
  --bmerge_cases.20.bed _cases.20.bim _cases.20.fam --make-bed --out 'conversion/_cases'
plink --noweb --bfile 'conversion/_cases' \
  --bmerge_cases.21.bed _cases.21.bim _cases.21.fam --make-bed --out 'conversion/_cases'
plink --noweb --bfile 'conversion/_cases' \
  --bmerge_cases.22.bed _cases.22.bim _cases.22.fam --make-bed --out 'conversion/_cases'
plink --noweb --bfile 'conversion/_cases' \
  --bmerge_cases.2.bed _cases.2.bim _cases.2.fam --make-bed --out 'conversion/_cases'
plink --noweb --bfile 'conversion/_cases' \
  --bmerge_cases.3.bed _cases.3.bim _cases.3.fam --make-bed --out 'conversion/_cases'
plink --noweb --bfile 'conversion/_cases' \
  --bmerge_cases.4.bed _cases.4.bim _cases.4.fam --make-bed --out 'conversion/_cases'
plink --noweb --bfile 'conversion/_cases' \
  --bmerge_cases.5.bed _cases.5.bim _cases.5.fam --make-bed --out 'conversion/_cases'
plink --noweb --bfile 'conversion/_cases' \
  --bmerge_cases.6.bed _cases.6.bim _cases.6.fam --make-bed --out 'conversion/_cases'
plink --noweb --bfile 'conversion/_cases' \
  --bmerge_cases.7.bed _cases.7.bim _cases.7.fam --make-bed --out 'conversion/_cases'
plink --noweb --bfile 'conversion/_cases' \
  --bmerge_cases.8.bed _cases.8.bim _cases.8.fam --make-bed --out 'conversion/_cases'
plink --noweb --bfile 'conversion/_cases' \
  --bmerge_cases.9.bed _cases.9.bim _cases.9.fam --make-bed \
  --out 'conversion/conversion/_PBC_Complete'
```

Note, that a manual step is required above, as plink does not accept duplicate SNP names.

Merging cases with controls

Next run the R script `liftOver.R` to align SNP positions. Pathes in the script might have to be adapted. The output files will be put into the `conversion` subfolder which might have to be created manually.

Merge the files with, adapt the pathes, if necessary.

```
export CASES=conversion/_PBC_Complete
export CONTROLS=conversion/_PLINK_format_illumina_58C
plink --noweb --bfile $CASES  --bmerge $CONTROLS.bed $CONTROLS.bim $CONTROLS.fam --make-bed
```

Filtering genotypes

Finally, filtering steps are performed as described in the paper.

```
# <p> loci
plink --noweb --bfile pbc_merged --geno 0.02 --make-bed --out pbc_merged_miss
# <p> individuals
plink --noweb --bfile pbc_merged_miss --mind 0.02 --make-bed --out pbc_merged_missi
# <p> maf
plink --noweb --bfile pbc_merged_missi --maf 0.15 --make-bed --out pbc_merged_maf15
# <p> LD pruning
plink --noweb --bfile pbc_merged_maf15 --indep 50 5 2
plink --noweb --bfile pbc_merged_maf15 --extract plink.prune.in \
  --make-bed --out pbc_maf15_vif2
```

The final file `pbc_maf15_vif2` can be used to reproduce the analyses. This path is coded into the analysis scripts and has to be changed if another name was chosen.
