# Supplementary material for: Powerful testing via hierarchical linkage disequilibrium in haplotype association studies
Source: Biom J. 2019 Jan 28;61(3):747–68. doi: 10.1002/bimj.201800053 (PMC6637384; doi:10.1002/bimj.201800053)
Supplement: Supplementary file 2 — Supporting Information [file BIMJ-61-747-s002.pdf]

Biometrical Journal **0** (0) 0, zzz–zzz / DOI: 10.1002/

Powerful testing via hierarchical linkage disequilibrium in haplotype association studies - Supplementary material

Brunilda Balliu[1], **Jeanine J. Houwing-Duistermaat** [2], and **Stefan Böhringer** [3*]

[1] Department of Pathology, Stanford University School of Medicine, 300 Pasteur Drive Stanford, CA 94305-5324, [2] School of Mathematics, University of Leeds, Leeds, UK, [3] Department of Biomedical Data Sciences, Leiden University Medical Center, Leiden, The Netherlands

1 Tower criterion

In order to reduce the number of false positive, SNPs were filtered by a weighted local correlation between location and log P-value. To this end, all SNPs within a window of 5×10^5 base pairs were determined and a weighted correlation between distance from the key SNP and log P-values was correlated. The weights were calculated by using an exponential decay function $\exp -D\zeta$ with D measured in base pairs and a decay constant $\zeta = 10^{-5}$. Window size and decay constant were not systematically investigated. A few combinations were tried to achieve reasonable results. The weighted correlation was computed using R-packages *wCorr*. As this packages does not compute P-values for the correlation, unweighted Pearson-correlation was used to assess the strength of correlation. Filtering was based both on the weighted correlation (*tower score*) and the P-value. We used a positive correlation and P-value < 0.1 in the paper.

The rationale for using such a filtering is that due to the overlap in a moving window analysis, P-values of haplotype tests should be correlated even if the underlying SNPs are uncorrelated. Adding to this the abundant positive correlation between SNPs most positive findings should exhibit local spatial correlation.

2 Haplotype analyses spanning two loci

A QQ-plot of p-values of all tests for analyzing two loci simultaneously are shown in figure 3. Before plotting, P-values were inflation corrected. This means that P-values were back-transformed to χ_1^2 test statistics, divided by the median test statistic and transformed back to P-values, making the median P-value equal 0.5. The Manhattan plot for the p-values is shown in figure **??**. Table 1 lists loci for which at least three tests showed a P-value $< 5 \times 10^{-8}$,

Table 1: Positions (*chr, pos*) and p-values for tests for which at least three tests reached a p-value $< 5 \times 10^{-8}$ when haplotypes span two loci. Numbers after BU, TD denote orders, *e.g.* 2 for $P = \{2\}$. P-values were filtered by a positive *tower score* and a P-value for the local correlation < 0.1.

chr	pos	kim	BU.Full	HLDmin	iterHLD	haplo.stats	TD.2	BU.1	marginal
1	31008523	1.8e-249	3.3e-309	3.3e-309	6.5e-309	8.6e-171	7.1e-311	-	-
1	31032093	1.7e-28	2.9e-33	2.9e-33	5.8e-33	-	7.4e-35	-	-
1	31033624	1.5e-185	1.1e-224	1.1e-224	2.2e-224	1.6e-08	2.7e-226	-	-
1	90440693	1.8e-32	-	1.2e-33	2.3e-33	6.0e-33	-	-	-
1	90481133	9.6e-185	-	2.1e-224	4.2e-224	5.4e-165	-	-	-
5	59580410	2.2e-09	2.6e-10	2.6e-10	5.2e-10	-	-	-	-
5	122166044	-	-	4.1e-151	8.2e-151	2.1e-119	-	-	-
5	153251949	-	3.3e-189	3.3e-189	6.5e-189	2.3e-140	-	-	-
6	26564048	1.7e-251	5.5e-302	5.5e-302	1.1e-301	5.5e-172	-	-	-

* Correspondence: correspondence@s-boehringer.org, +31 71 5269743

© 0 WILEY-VCH Verlag GmbH & Co. KGaA, Weinheim

7	88709037	-	3.4e-140	-	-	6.7e-145	4.5e-90	-	-
7	149498823	2.9e-100	3.2e-09	6.3e-10	6.3e-10	1.0e-11	-	6.3e-10	-
7	149504002	-	4.6e-11	1.1e-11	1.1e-11	1.0e-13	-	1.1e-11	2.5e-13
12	68141467	-	8.8e-79	8.8e-79	1.8e-78	1.1e-68	-	-	-
12	99342932	5.1e-56	1.0e-59	1.0e-59	2.0e-59	1.2e-54	-	-	-
13	82025812	8.4e-246	1.1e-320	1.1e-320	2.1e-320	-	1.5e-323	-	-
16	60188415	8.5e-10	8.0e-11	8.0e-11	1.6e-10	1.3e-10	-	-	-
17	66951514	-	1.1e-35	1.1e-35	2.2e-35	-	-	-	-
18	56526010	-	2.5e-162	2.5e-162	5.0e-162	3.3e-126	9.9e-165	-	-
19	63603580	4.5e-17	6.6e-19	6.6e-19	1.3e-18	3.1e-18	8.9e-21	-	-

3 Haplotype analyses spanning three loci

Table 2: Positions (*chr, pos*) and p-values for tests for which at least three tests reached a p-value $< 5 \times 10^{-8}$ when haplotypes span three loci. Numbers after BU, TD denote orders, *e.g.* 12 for $P = \{1, 2\}$. P-values were filtered by a positive *tower score* and a P-value for the local correlation < 0.1.

chr	pos	TD.23	BU.12	BU.Full	kim	TD.3	haplo.stats	HLDmin	iterHLD	BU.1	marginal
1	31024793	-	2.7e-89	-	-	-	-	8.9e-95	2.7e-94	-	-
1	37189748	-	2.3e-51	7.4e-51	-	-	1.4e-39	1.1e-51	1.1e-51	1.1e-51	-
2	12008889	2.3e-69	1.1e-67	7.0e-67	-	-	-	-	-	-	-
2	27044977	-	-	-	4.7e-138	1.0e-10	1.1e-188	-	-	-	-
2	38599502	2.1e-59	3.4e-50	6.3e-57	-	-	-	-	-	-	-
2	140376310	8.4e-139	1.1e-122	2.0e-136	-	-	-	2.0e-136	-	-	-
3	124354616	-	-	2.6e-321	1.4e-214	5.4e-20	1.5e-138	-	-	-	-
3	175898039	3.8e-75	1.3e-40	8.9e-73	-	-	-	8.9e-73	2.7e-72	-	-
3	177492211	1.6e-253	5.0e-252	4.7e-251	-	-	-	5.0e-252	1.0e-251	-	-
4	168447627	-	3.0e-232	6.2e-241	-	1.5e-11	-	6.2e-241	1.8e-240	-	-
4	168473476	-	-	2.0e-98	-	7.9e-13	-	2.0e-98	5.9e-98	-	-
5	166681168	3.7e-210	1.1e-203	9.1e-208	-	9.2e-07	-	-	2.7e-207	-	-
6	167442290	1.1e-139	5.6e-125	-	-	3.1e-15	-	-	-	-	-
7	56114450	1.2e-224	7.6e-169	-	-	-	6.6e-53	-	-	-	-
7	149394699	-	8.3e-08	2.4e-07	-	-	2.0e-10	3.7e-09	3.7e-09	3.7e-09	-
7	149504002	-	-	-	3.7e-102	-	2.3e-13	-	-	-	2.5e-13
11	71529215	6.1e-39	1.5e-31	8.9e-37	-	-	-	8.9e-37	2.7e-36	-	-
12	44329880	4.0e-58	2.7e-54	2.1e-56	-	-	-	2.1e-56	6.3e-56	-	-
12	125246299	5.0e-84	7.0e-54	1.2e-81	-	6.3e-31	-	-	-	-	-
12	125264114	5.9e-112	8.9e-57	1.4e-109	-	2.2e-56	-	-	-	-	-
13	28446805	4.2e-202	7.0e-200	6.1e-199	-	-	-	-	-	-	-
14	52938344	1.9e-23	6.4e-16	4.6e-23	-	-	-	4.6e-23	-	-	-
14	102632948	-	3.4e-07	7.4e-07	-	-	-	4.0e-08	4.0e-08	4.0e-08	-
15	53083156	-	3.0e-92	3.8e-100	-	-	-	3.8e-100	1.1e-99	-	-
17	27868497	-	-	-	-	-	2.7e-47	9.0e-53	2.6e-52	-	-
17	42299827	-	2.0e-51	9.7e-88	-	-	-	9.7e-88	2.9e-87	-	-
17	42306776	-	4.1e-266	1.3e-271	-	2.7e-08	-	1.3e-271	3.8e-271	-	-
17	51463256	-	1.2e-22	1.1e-27	-	1.6e-07	-	-	-	-	-
18	45483250	-	1.9e-86	3.4e-108	-	-	-	3.4e-108	1.0e-107	-	-
19	54583931	-	2.6e-57	9.6e-59	2.7e-53	-	1.2e-07	9.6e-59	2.9e-58	-	-
23	71225646	1.4e-10	-	8.3e-57	-	-	6.1e-12	7.1e-57	-	-	-

© 0 WILEY-VCH Verlag GmbH & Co. KGaA, Weinheim

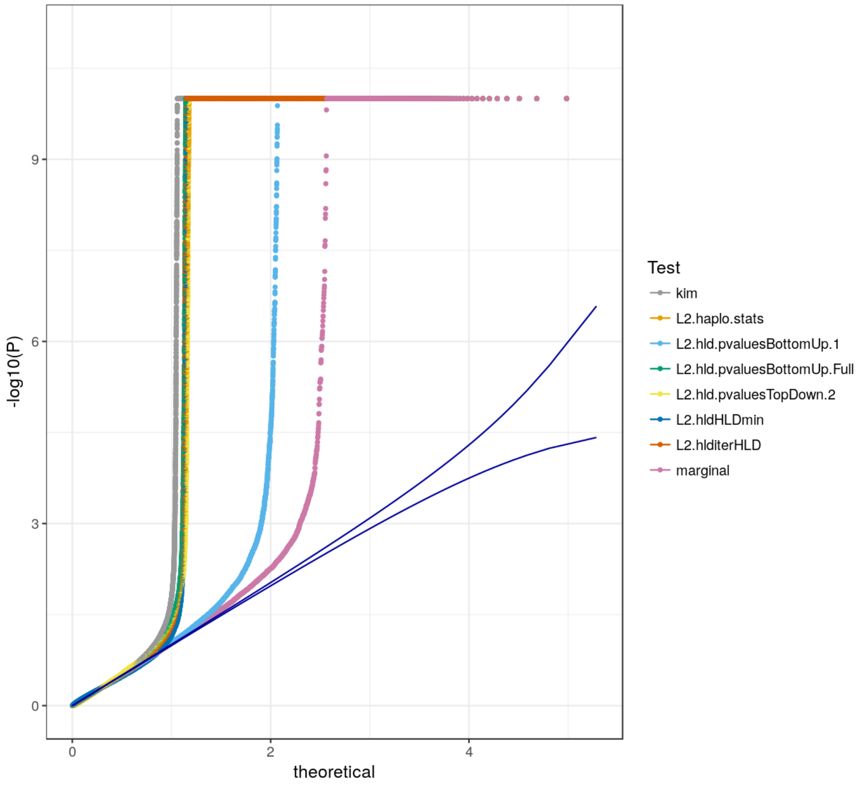

Figure 1 QQ plot for all two locus tests, including, for reference the marginal test. P-values were inflation-corrected before plotting (see text). Blue lines represent point-wise confidence limits for ordered P-values.

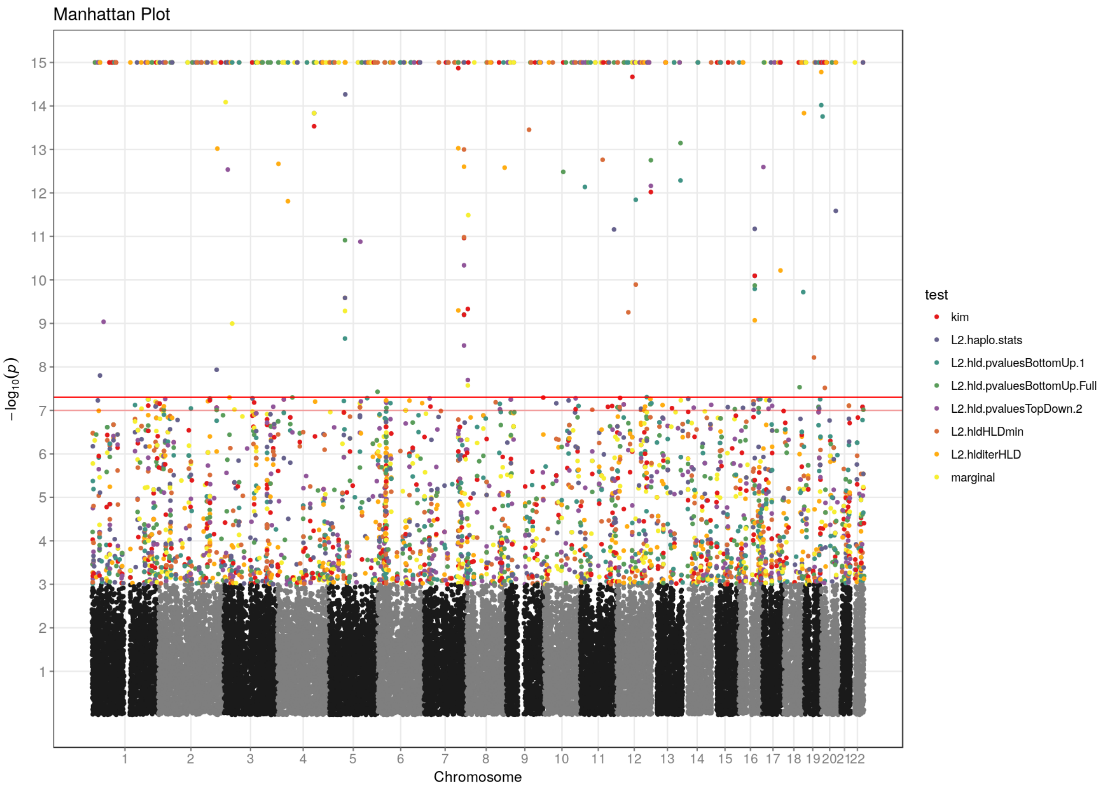

Figure 2 Manhatten plot of P-values for all test when haplotypes span **two** loci. Colors are applied to P-values $< 10^{-3}$. P-values $< 5 \times 10^{-8}$ were filtered by the *tower criterion*. P-values $< 10^{-15}$ were truncated.

© 0 WILEY-VCH Verlag GmbH & Co. KGaA, Weinheim

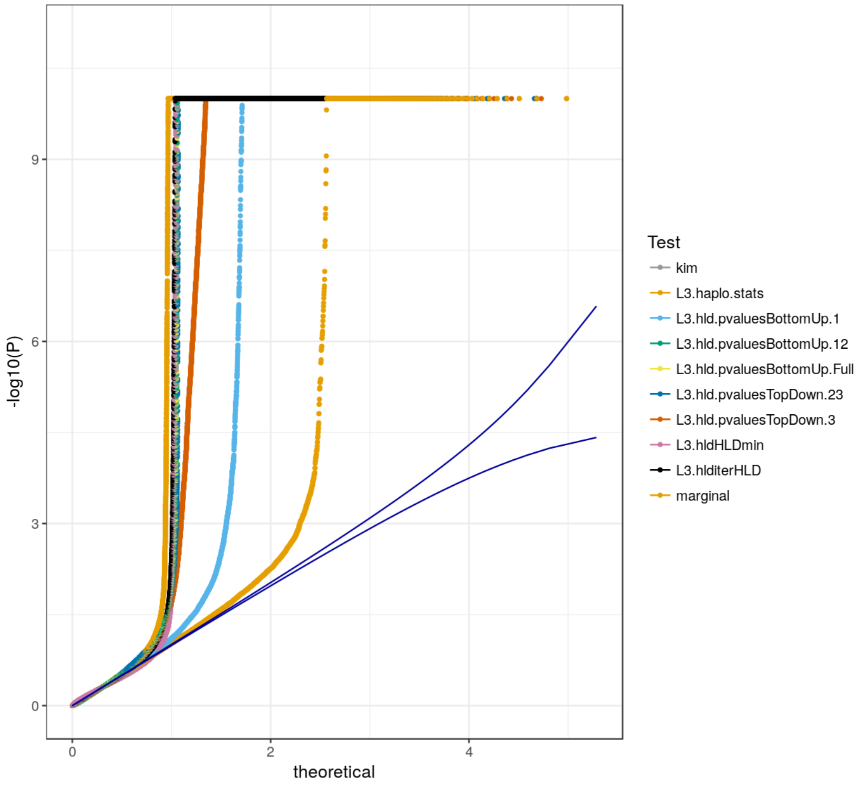

Figure 3 QQ plot for all three locus tests, including, for reference the marginal and kim tests. P-values were inflation-corrected before plotting (see text). Blue lines represent point-wise confidence limits for ordered P-values.

© 0 WILEY-VCH Verlag GmbH & Co. KGaA, Weinheim

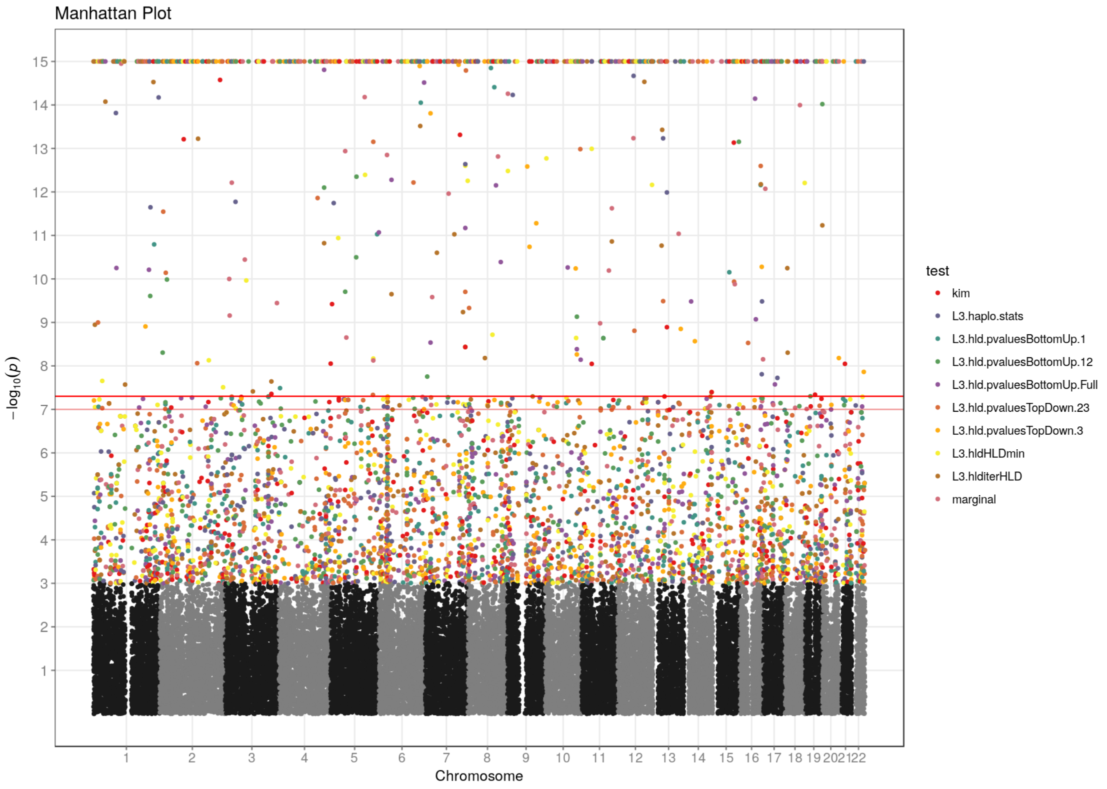

Figure 4 Manhatten plot of P-values for all test when haplotypes span **three** loci. Colors are applied to P-values $< 10^{-3}$. P-values $< 5 \times 10^{-8}$ were filtered by the *tower criterion*. P-values $< 10^{-15}$ were truncated.

© 0 WILEY-VCH Verlag GmbH & Co. KGaA, Weinheim
